# Effectiveness of telephone-based aftercare case management for adult patients with unipolar depression compared to usual care: A randomized controlled trial

**DOI:** 10.1371/journal.pone.0186967

**Published:** 2017-10-27

**Authors:** Laura Kivelitz, Levente Kriston, Eva Christalle, Holger Schulz, Birgit Watzke, Martin Härter, Lutz Götzmann, Harald Bailer, Sabine Zahn, Hanne Melchior

**Affiliations:** 1 Department of Medical Psychology, University Medical Center Hamburg-Eppendorf, Hamburg, Germany; 2 Clinical Psychology and Psychotherapy Research, Institute of Psychology, University of Zurich, Zurich, Switzerland; 3 Department of Psychosomatic Medicine and Psychotherapy, Segeberg Hospital, Bad Segeberg, Bad Segeberg, Germany; 4 Luisenklinik - Zentrum für Verhaltensmedizin, Bad Dürrheim, Germany; 5 Fachklinik für Psychosomatische Medizin und Psychotherapie, MediClin Seepark Klinik, Bad Bodenteich, Germany; Wayne State University, UNITED STATES

## Abstract

**Background:**

Patients with depression often have limited access to outpatient psychotherapy following inpatient treatment. The objective of the study was to evaluate the long-term effectiveness of a telephone-based aftercare case management (ACM) intervention for patients with depression.

**Methods:**

We performed a prospective randomized controlled trial in four psychotherapeutic inpatient care units with N = 199 patients with major depression or dysthymia (F32.x, F33.x, F34.1, according to the ICD-10). The ACM consisted of six phone contacts at two-week intervals performed by trained and certified psychotherapists. The control group received usual care (UC). The primary outcome was depressive symptom severity (BDI-II) at 9-month follow-up, and secondary outcomes were health-related quality of life (SF-8, EQ-5D), self-efficacy (SWE), and the proportion of patients initiating outpatient psychotherapy. Mixed model analyses were conducted to compare improvements between treatment groups.

**Results:**

Regarding the primary outcome of symptom severity, the groups did not significantly differ after 3 months (*p* = .132; ES = -0.23) or at the 9-month follow-up (*p* = .284; ES = -0.20). No significant differences in health-related quality of life or self-efficacy were found between groups. Patients receiving ACM were more likely to be in outpatient psychotherapy after 3 months (OR: 3.00[1.12–8.07]; *p* = .029) and 9 months (OR: 4.78 [1.55–14.74]; *p* = .006) than those receiving UC.

**Conclusions:**

Although telephone-based ACM did not significantly improve symptom severity, it seems to be a valuable approach for overcoming treatment barriers to the clinical pathways of patients with depression regarding their access to outpatient psychotherapy.

## Background

Depression is one of the most prevalent mental disorders [1] and is associated with a significant personal, social and economic burden [2, 3]. Despite the high 12-month prevalence of 7.7% in Germany [4], only approximately 50% of these patients receive appropriate treatment [5], cf. [6]. Barriers to patients’ pathways to treatment include communication and coordination problems between different services and providers [7–10]. These gaps in continuity of care frequently occur in the transition of patients from inpatient treatment to outpatient aftercare. Treatment barriers that result from a lack of integration of the different steps involved in care can emerge both at the systemic level (e.g., long waiting lists for outpatient psychotherapy) and at the individual level (e.g., insufficient patient awareness of available treatment options) [8, 11], cf. [6].

There is evidence supporting the effectiveness of inpatient treatment, but the effects of treatment often decrease after discharge [12, 13]. Therefore, follow-up outpatient psychotherapy is often indicated and recommended for patients with depressive disorders [14]. Here, the aims are to consolidate the treatment outcomes of inpatient treatment and stabilize positive treatment effects [15]. Depressive disorders are particularly highly recurrent [16, 17], and the risk of further episodes increases with each additional episode (cf. [6]). To ensure continuity of care, innovative low-threshold aftercare concepts, e.g., online- or telephone-based aftercare case management interventions, could be beneficial [7, 18, 19]. Case management is a patient-centred and situation-based approach that comprises systematic tracking and support of patients by a case manager. The primary goal is to coordinate and integrate services across treatment settings by providing self-management support and follow-up for patients [7], cf. [6]. Advantages of the telephone-based case management approach include the widespread availability, easy usage and ability to maintain personal contact regardless of place of residence. Research on the effectiveness of case management programmes for patients with depression has shown positive effects on symptom severity, quality of life, and patient satisfaction [20, 21]. Most of the studies were conducted in primary care and showed significant clinical benefits of case management [21–26] with only a modest increase in health services costs [24, 27].

Although most studies investigating the effectiveness of case management-based aftercare for patients with different mental disorders have shown positive effects on health care utilization and quality of life [28–30], the findings regarding symptom burden are mixed. In some trials, positive effects on depressive symptoms have been found [18, 30], while in others, telephone case management did not successfully reduce depressive symptom severity [28, 29, 31]. Furthermore, in the German health care system, case management programmes for depression have been poorly investigated.

## Methods

### Trial design

The study was conducted as a prospective multicentre randomized controlled trial (RCT). The patients who received telephone-based ACM were compared to those who received UC regarding the primary and secondary outcomes. Measurements were performed at three time points in each group. Baseline measures were taken at discharge from inpatient treatment (t_1_), which marked the beginning of the intervention for the ACM group. Follow-up measures were obtained 3 months after discharge (t_2_), which was the end of the intervention, and 9 months after discharge (t_3_), which was 6 months after the end of the intervention. The follow-up measures were completed in June 2015. Participants received follow-up paper-pencil questionnaires by post and were reminded twice by their therapists via phone calls and once via reminder letter if they were not accessible.

This study aimed to evaluate the long-term effectiveness of aftercare case management (ACM) following inpatient treatment for patients with depression. The primary outcome was symptom severity at 9 months after discharge from inpatient treatment (t_3_). Additionally, measurements 3 months after discharge (t_2_) were obtained. The secondary outcomes were health-related quality of life, self-efficacy, and initiation of outpatient psychotherapy at t_2_ or t_3_. We hypothesized that patients in the ACM group would exhibit significantly better outcomes than patients in a control group receiving usual care (UC).

### Randomization

After written informed consent was obtained, patients were randomly assigned to the ACM or the UC group. The patients were randomized using randomly varying block sizes (between 2 and 8) to ensure concealment and comparable group sizes. The randomization was stratified by the participating clinical units and was conducted at the individual level at the study centre (University Medical Center Hamburg-Eppendorf) one week before the beginning of the intervention. The allocation schedule was created with the “ralloc” command of STATA version 12 (StataCorp LLC, College Station, TX) by a researcher who did not participate in patient recruitment. The therapists were informed of the randomization outcomes via email prior to the last psychotherapeutic session of the patients’ inpatient treatment. Thus, the therapists were able to inform the patients about their group assignment prior to discharge.

### Study setting and participant recruitment

Participants were recruited consecutively by their psychotherapists during their treatment in four psychotherapeutic inpatient units in Germany (St. Franziska-Stift Bad Kreuznach, MediClin Seepark Klinik Bad Bodenteich, Segeberger Kliniken Gruppe Bad Segeberg and Luisenklinik Bad Dürrheim) from October 2012 to October 2014. Inpatient treatment consisted of at least one session of individual psychotherapy and two sessions of group psychotherapy per week. Additionally, patients received psychoeducation, exercise and relaxation training. All of the therapists had obtained a master’s degree in clinical psychology or medicine at a minimum and had completed or were in advanced standing in a multiyear postgraduate professional psychotherapy training programme.

### Inclusion an exclusion criteria

The inclusion criteria were diagnosed depression (F32.x, F33.x, F34.1, according to the ICD-10) [32] and a recommendation of outpatient psychotherapy after discharge from the inpatient unit. Patients were required to be at least 18 years old. The diagnoses were validated with the Mini-DIPS diagnostic interview [33], which is a short version of the Diagnostic Interview for Mental Disorders (DIPS) [34]. Patients who had received concurrent outpatient psychotherapeutic treatment before their admission that was planned to be continued after inpatient treatment were excluded from the study. Further exclusion criteria included an acute risk of suicide, acute psychosis or psychotic symptoms, insufficient German language skills, and an inpatient treatment duration of less than three days. Prior to participation, the patients were informed of the study by their therapists.

The study was approved by the responsible local Ethics Committee of the Chamber of Physicians in Hamburg in February 27^th^, 2012 (Ref. Nr. PV4004) and was conducted according to the principles of the Declaration of Helsinki (2013 version). The study was registered in the German Clinical Trials Register (NCT02044913). The study protocol was published in Kivelitz et al. [6]. Due to organisational changes in the research project shortly before the start of the recruitment we put great efforts into avoiding a delayed start of the data collection in the cooperating inpatient units, which resulted in retrospective study registration and a delayed publication of our study protocol. The authors confirm that all ongoing and related trials for this intervention are registered.

### Intervention

ACM is based on the concept of case management [7] and aims to support the patient in finding and organizing his or her individual aftercare treatment following inpatient treatment. After discharge, patients in the ACM-group received systematic aftercare case-management, consisting of six aftercare phone contacts at intervals of two weeks performed by their inpatient treatment therapists. The responsibility of the therapists was to support and guide the patients in matters related to making plans and generating goals regarding the coordination of their aftercare treatment. The patients were motivated and encouraged in terms of their own empowerment to become active in organizing their own aftercare treatment. Therapists provided feedback by monitoring the steps taken towards goal achievement. During ACM, and in contrast to therapeutic interventions, the treatment of patients’ disorder-specific complaints was not the primary focus. The main content of the phone contacts included the patients’ needs and problems associated with their aftercare as well as supportive consultation from the therapist.

Prior to initiating the ACM, the therapists were trained in their role as a case manager and received a detailed manual that provided guidelines for the phone contacts. This manual contained detailed instructions for the therapists but allowed sufficient freedom to tailor the aftercare to the patients’ needs and the individual context. The manual included descriptions of the aims of aftercare, the processes involved in the phone contacts and instructions for dealing with specific situations. The phone contacts were designed to last 20 to 30 minutes each. After each phone contact, the therapists completed a self-developed questionnaire that included process documentation of the duration and content of the contact. After discharge, besides ACM, the patients utilized health care services as needed and desired.

### Control group

After inpatient treatment, patients in the UC group received usual care that did not involve systematic ACM. The patients in the UC group did not receive any contact with their therapist from the clinic after being discharged from inpatient treatment. After discharge, the patients utilized health care services as needed and desired.

### Outcome measures

The primary outcome measure was symptom severity, which was assessed at all time points (t_1_, t_2_, t_3_) using Beck’s Depression Inventory (BDI-II) [35]. The BDI-II consists of 21 items that are rated on a four-point Likert scale and yields a total sum score (range: 0 to 63). Higher scores indicate higher symptom severity. The secondary outcomes were health-related quality of life, self-efficacy and initiation of outpatient psychotherapy at t_2_ and t_3_. Health-related quality of life was measured with the EuroQol-5D [36] and the Short-Form 8 Health Survey (SF-8) [37]. The EuroQol-5D is a valid generic instrument that measures health-related quality of life in five dimensions; i.e., mobility, self-care, usual activities, pain/discomfort and anxiety/depression. Health-related quality of life is also assessed with a visual analogue scale (range: 0 to 100; higher ratings indicate higher quality of life). Health states are converted into a weighted health state index (full health has a value of 1 and dead a value of 0.) The SF-8 is a short version of the SF-36 [38] and allows for the calculation of scores of physical and mental health (range: 0 to 100; higher scores indicate better statuses). In the present study, we refer to the mental health dimension. To assess self-efficacy, the German version of the General Self-Efficacy Scale (SWE) [39] was used. This scale comprises 10 four-point Likert-scale items and yields a summed score (range: 10 to 40; higher scores indicate greater general self-efficacy). Initiating or starting outpatient psychotherapy at follow-up was assessed with self-report items asking the patients at t_2_ and at t_3_ if they were currently in or on the waiting list for outpatient psychotherapy.

### Sample size calculation

We conducted a power calculation for the planned ANCOVA analyses by using the tool G*Power [40]. To detect a moderate effect (Cohen’s d = .5) between the intervention and control groups in terms of symptom severity with a power of 80% and level of significance, α, of 0.05, a sample size of n = 96 (48 per group) was required. Based on an expected dropout rate of approximately 50% from baseline (t_1_) to the 9-month follow-up (t_3_), we aimed to include a sample of at least n = 192 (96 per group) to perform the completer analysis with a sufficient sample size (see [6]). In the initial study plan, patients with anxiety disorders were also a focus, and thus, anxiety symptom severity was also originally designed to be an additional outcome [6]. The recruitment of patients with anxiety proved difficult, which led to a very low number of patients with anxiety. Therefore, we decided to focus solely on the group of depressive patients. The required sample size was still sufficient for the effectiveness analyses.

### Statistical analyses

Comparisons between the ACM and the UC group were calculated to test whether patients in the ACM group exhibit better treatment outcomes (dependent variables) than those in the UC group over time, using mixed model analyses with repeated measurements. All models included the variables ‘group’ (ACM and UC), ‘measurement time point’ (t_1_, t_2_ and t_3_), their interaction term ‘group × time’, ‘trial site’, and ‘ongoing process of pension application’ as fixed effects. Groups differed substantially regarding the process of pension application at baseline. As it can be assumed that this variable is strongly associated with the outcome (people who wish to retire might be less motivated to recover, e.g. by starting a psychotherapy), it was retained as a covariate in the effectiveness analyses. To model interindividual differences, random intercept was also included in the models. Following the intention-to-treat (ITT) approach, we analysed all randomized participants in the primary analysis. Additionally, sensitivity analyses were conducted that included only participants who had completed all measurements (completer analyses). We also performed analyses of covariance (ANCOVA) based on the ITT data as well as on the completer data with ‘group’, ‘trial site’, ‘initial symptom severity’ and ‘ongoing process of pension application’ as independent variables. Additionally, we conducted drop-out analyses at t_2_ and t_3_ (*t*- and χ^2^-tests) to compare the demographic and clinical baseline characteristics of the participants who completed the follow-up assessments (completers) and those who were lost to follow-up (non-completers). For group comparisons, we calculated standardized between-group effect sizes (Cohen’s *d*) by dividing the model-based difference of the group means by the observed standard deviation of the UC group.

Regarding the proportion of patients who started outpatient psychotherapy or were on the waiting list for outpatient psychotherapy at follow-up, binomial logistic regression analyses were conducted for t_2_ and t_3_ with gender, age, initial symptom severity (BDI-II scores at t_1_) and ongoing process of pension application as covariates and group as the predictor. We conducted completer analyses as main analyses because there was no theoretical or empirical basis justifying data imputation for ‘treatment status’. However, we performed two different ITT sensitivity analyses. In the ‘ITT best case sample’, missing data in the ACM group were imputed as ‘in psychotherapy’ and as ‘not in psychotherapy’ in the UC-group; in the ‘ITT worst case sample’, the imputation was performed with the conditions switched. The analyses were conducted with SPSS PASW 18 (SPSS Inc., Chicago, IL).

Because of its advantages in managing missing values, we preferred the mixed model approach over the ANCOVA analyses initially planned [6] to analyse our data. Missing data were not imputed explicitly, since mixed model analyses provide unbiased estimates under the assumption that data are missing at random conditional on the variables in the model.

## Results

### Participants

**Fig 1:**
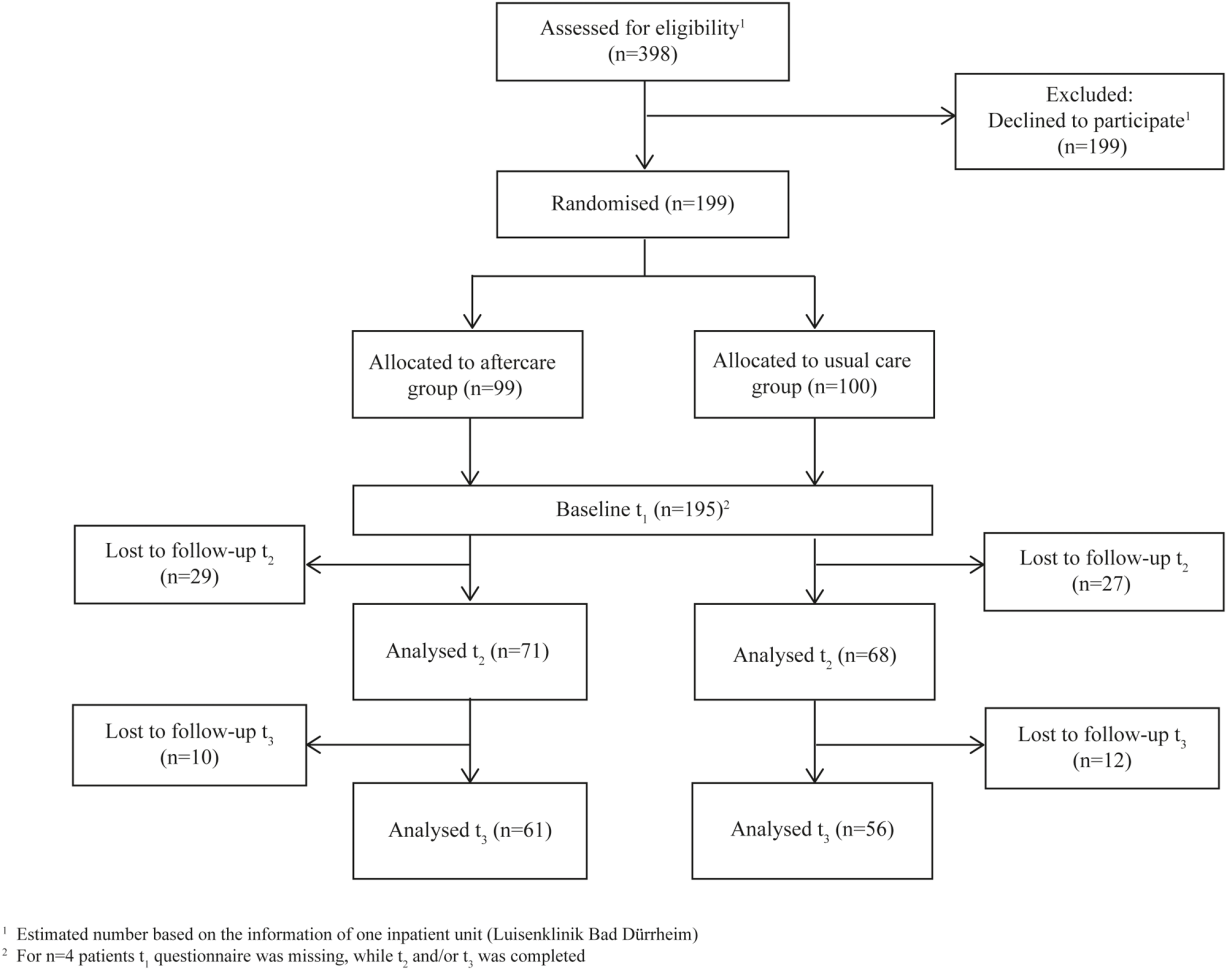
Flow of participants through the trial.

Fig 1 illustrates the flow of participants through the study. In total, 398 patients were recruited consecutively after being screened for inclusion criteria. About half of them declined to participate. In total, 199 participants were enrolled in the study.

Overall, 33 registered psychotherapists or psychologists in advanced psychotherapy training from the four cooperating clinical units participated (inpatient unit 1: n = 10, inpatient unit 2: n = 3, inpatient unit 3: n = 5, inpatient unit 4: n = 15 therapists). The majority of the patients (78.5%) received six phone contacts, 5.4% received four phone contacts, 6.5% three contacts, 2.2% two contacts and 7.5% only one phone contact. The mean duration of the phone contacts, which took place every two weeks, was 22.7 minutes (SD = 7.1).

Three months after inpatient treatment (t_2_), 139 participants completed the assessment (drop-out rate of 30%), and 117 completed the 9-month follow-up (t_3_) (drop-out rate of 41%), with drop-out rates of 28.3% in the ACM group at t_2_ and 38.4% at t_3_ and of 32% in the UC group at t_2_ and 44% at t_3_. Drop-out analyses at t_2_ and t_3_ did not show any statistically significant baseline differences between the completers and non-completers.

Participant characteristics at baseline are shown in Table 1. Participants were predominately female, with an average age of 44 years. The majority of the participants had a high educational level and was employed full- or part-time. Almost all participants (99%) had one or more mental disorder diagnoses in addition to the depression diagnosis.

**Table 1:** Baseline demographic and clinical characteristics of the study participants (n = 199).

	Total n = 199	ACM group n = 99	UC group n = 100
**Demographic characteristics**			
Age at baseline in years, M (SD)	44.0 (11.0)	43.9 (10.7)	44.1 (11.4)
Female, n (%)	144 (73.8)	69 (72.6)	75 (75.0)
Married/partnership, n (%)	126 (66.7)	67 (72.0)	59 (61.5)
High educational level^a^, n (%)	125 (64.4)	64 (68.1)	61 (61.0)
Full- or part-time-employment, n (%)	121 (63.7)	57 (60.6)	64 (66.7)
Sick leave in weeks^b^, M (SD)	12.5 (5.6)	13.1 (9.7)	11.9 (9.5)
Ongoing process of pension application, n (%)	12 (6.5)	9 (10.2)	3 (3.1)
**Treatment-related characteristics**			
Previous inpatient pretreatment because of current complaints, n (%)	66 (34.6)	32 (34.4)	34 (34.7)
Outpatient pretreatment (psychotherapy)^b^, n (%)	36 (18.9)	17 (18.1)	19 (19.8)
Use of antidepressants^c^, n (%)	98 (52.7)	51 (57.3)	47 (48.5)
**Health-related characteristics**			
Symptom severity (BDI-II), M (SD)	23.1 (13.2)	23.5 (13.5)	22.6 (12.9)
Health-related quality of life (EuroQol-5D), M (SD)	0.63 (0.22)	0.61 (0.23)	0.64 (0.21)
Health-related quality of life (SF-8), M (SD)	36.4 (11.6)	37.1 (12.0)	35.6 (11.2)
Self-efficacy (SWE), M (SD)	12.8 (6.7)	12.8 (6.5)	12.7 (7.0)
**Diagnoses and comorbidities**			
Psychiatric comorbidities^d^ (one or more psychiatric diagnoses other than depression), n (%)	196 (99.0)	97 (98.0)	99 (100)
**Comorbidities**			
Anxiety disorders, (ICD-10 F40–41, F48), n (%)	49 (24.7)	25 (25.3)	24 (24.2)
Somatoform disorders (ICD-10 F45), n (%)	16 (8.1)	8 (8.1)	8 (8.1)
Adjustment disorders (ICD-10 F43.2), n (%)	13 (6.6)	6 (6.1)	7 (7.1)
Eating disorders (ICD-10 F50), n (%)	9 (4.5)	4 (4.0)	5 (5.1)
Personality disorders (ICD-10 F60-F61), n (%)	9 (4.5)	7 (7.1)	2 (2.0)
**Trial site**			
Inpatient unit 1, n (%)	44 (22.1)	20 (20.2)	24 (24.0)
Inpatient unit 2, n (%)	19 (9.5)	11 (11.1)	8 (8.0)
Inpatient unit 3, n (%)	103 (51.8)	54 (54.5)	49 (49.0)
Inpatient unit 4, n (%)	33 (16.6)	14 (14.1)	19 (19.0)

Results are expressed as the mean and standard deviation, M (SD), or numbers with percentages in parentheses, n (%).

^a^ Above secondary general school (more than nine years of education in the German system)

^b^ Within six months prior to admission.

^c^ Within four weeks prior to admission.

^d^ Known for n = 198 participants.

### Primary and secondary outcomes

Table 2 illustrates the results of the mixed model analyses based on the ITT data.

**Table 2:** Results of mixed-model analyses based on the ITT data regarding the primary and secondary outcomes.

Outcome	Means				Estimated mean difference (95% CI)	*p*	ES Cohen’s d
	Observed		Estimated				
	M (SD)		M (SE)				
Whole sample	ACM	UC	ACM	UC			
**Primary Outcome**							
**BDI-II**							
*Baseline (t*_*1*_*)*	23.5 (13.5)	22.6 (12.9)	25.3 (2.3)	25.5 (2.3)	-.21 (-3.71 to 4.12)	.917	-0.02
*3 months (t*_*2*_*)*	21.9 (13.4)	24.6 (14.1)	24.9 (2.2)	28.2 (2.4)	-3.27 (-0.99 to 7.54)	.132	-0.23
*9 months (t*_*3*_*)*	20.0 (13.8)	21.5 (12.0)	23.4 (2.3)	25.8 (2.4)	-2.44 (-2.03 to 6.90)	.284	-0.20
**Secondary Outcomes**							
**SF-8**							
*Baseline (t*_*1*_*)*	37.1 (12.0)	35.6 (11.2)	35.6 (1.7)	33.9 (1.8)	1.69 (-5.08 to 1.70)	.328	0.15
*3 months (t*_*2*_*)*	37.5 (10.9)	34.9 (11.5)	35.6 (1.9)	32.8 (2.0)	2.84 (-6.72 to 1.04)	.151	0.24
*9 months (t*_*3*_*)*	38.8 (11.2)	36.6 (10.9)	36.6 (1.9)	34.6 (2.1)	2.12 (-6.32 to 2.07)	.320	0.18
**EQ-5D**							
*Baseline (t*_*1*_*)*	0.61 (0.23)	0.64 (0.21)	0.55 (0.04)	0.57 (0.04)	.015 (-0.50–0.08)	.659	-0.10
*3 months (t*_*2*_*)*	0.63 (0.23)	0.61 (0.23)	0.57 (0.04)	0.54(0.04)	.025 (-0.10 to 0.05)	.492	0.13
*9 months (t*_*3*_*)*	0.63 (0.25)	0.65 (0.19)	0.56 (0.04)	0.57(0.04)	-.012 (-0.06 to 0.09)	.760	-0.05
**SWE**							
*Baseline (t*_*1*_*)*	12.8 (6.51)	12.7 (6.96)	12.6 (1.1)	12.3 (1.2)	0.36 (-2.36 to 1.64)	.724	0.04
*3 months (t*_*2*_*)*	14.3 (6.69)	12.9 (7.19)	13.7 (1.1)	12.2 (1.2)	1.48 (-3.65 to 0.70)	.183	0.21
*9 months (t*_*3*_*)*	14.8 (6.71)	14.2 (7.15)	14.4 (1.2)	12.7 (1.2)	1.65 (-3.92 to 0.61)	.152	0.24

Results are expressed as M (SD) = mean (standard deviation) and M (SE) = mean (standard error).

Overall, groups did not statistically significantly differ regarding the course of depressive symptom severity from baseline to nine months (interaction effect ‘group x time’: F_2, 248.433_ = 1.637; *p* = .197). Regarding the primary outcome of depressive symptom severity, the groups did not significantly differ at t_2_ (*p* = .132; ES = -0.23) or at t_3_ (*p* = .284; ES = -0.20). With respect to the SF-8, the EQ-5D and the SWE, patients receiving ACM and those in the UC group did not show statistically significant differences. The ANCOVA analyses (reported in S2 Table) largely confirm the reported findings.

The sensitivity analyses of the completer data (see Table 3) showed similar results as the ITT analyses. The difference between the ACM and UC groups regarding the primary outcome did not reach statistical significance at t_2_ (*p* = .079; ES = -0.35) and at t_3_ (*p* = .194; ES = -0.28). Regarding health-related quality of life (SF-8, EQ5D) and self-efficacy (SWE), no differences between the groups were found at either t_2_ or t_3_. The reported findings were largely confirmed by the ANCOVA analyses (reported in S3 Table).

**Table 3:** Results of mixed-model analyses based on completer data regarding the primary and secondary outcomes.

Outcome	Means				Estimated mean difference (95% CI)	*p*	ES Cohen’s d
	Observed		Estimated				
	M (SD)		M (SE)				
Total sample (n = 101)	ACM (n = 48)	UC (n = 53)	ACM (n = 48)	UC (n = 53)			
**Primary Outcome**							
**BDI-II**							
*Baseline (t*_*1*_*)*	21.7 (13.7)	21.3 (12.7)	25.8 (2.8)	27.0 (3.1)	-1.27 (-3.83 to 6.38)	.623	-0.09
*3 months (t*_*2*_*)*	21.0 (13.4)	23.5 (13.1)	24.7 (2.8)	29.3 (3.1)	-4.56 (-.54 to 9.7)	.079	-0.35
*9 months (t*_*3*_*)*	20.0 (13.5)	21.4 (12.0)	23.7 (2.8)	27.1 (3.1)	-3.37 (-1.73 to 8.47)	.194	-0.28
**Secondary Outcomes**							
**SF-8**							
*Baseline (t*_*1*_*)*	38.4 (12.2)	36.6 (10.6)	35.1 (2.2)	33.0 (2.4)	2.16 (-6.58 to 2.26)	.337	0.20
*3 months (t*_*2*_*)*	37.8 (11.1)	34.9 (11.3)	35.0 (2.2)	31.4 (2.4)	3.59 (-7.99 to 0.82)	.110	0.32
*9 months (t*_*3*_*)*	38.7 (11.2)	36.9 (10.8)	35.7 (2.2)	33.4 (2.4)	2.31 (-6.74 to 2.11)	.304	0.21
**EQ-5D**							
*Baseline (t*_*1*_*)*	0.64 (0.21)	0.64 (0.19)	0.56 (0.04)	0.53 (0.05)	0.02 (-0.11 to 0.06)	.573	0.16
*3 months (t*_*2*_*)*	0.65 (0.21)	0.63 (0.22)	0.58 (0.05)	0.53 (0.05)	0.05 (-0.13 to 0.03)	.248	0.23
*9 months (t*_*3*_*)*	0.62 (0.25)	0.65 (0.19)	0.55 (0.04)	0.56 (0.05)	-0.01 (-0.07 to 0.09)	.784	-0.05
**SWE**							
*Baseline (t*_*1*_*)*	12.8 (6.51)	12.7 (6.96)	12.1 (1.5)	12.6 (1.7)	-0.51 (-2.23 to 3.25)	.711	-0.07
*3 months (t*_*2*_*)*	14.3 (6.69)	12.9 (7.19)	14.3 (1.0)	12.6 (1.7)	1.64 (-4.38 to -1.10)	.239	0.24
*9 months (t*_*3*_*)*	14.8 (6.71)	14.2 (7.15)	14.6 (1.5)	13.0 (1.7)	1.56 (-4.29 to -0.19)	.264	0.22

Results are expressed as M (SD) = mean (standard deviation) and M (SE) = mean (standard error).

Table 4 illustrates the frequencies of patients who initiated outpatient psychotherapy. These values included patients who had already started outpatient psychotherapy and patients who were on the waiting list for psychotherapy at t_2_ and t_3_.

**Table 4:** Patients who initiated outpatient psychotherapy at t_2_ and t_3_ in the ACM and UC groups.

Secondary Outcome		Total n (%)	ACM group n (%)	UC group n (%)
Treatment status of outpatient psychotherapy (TSP): being in outpatient psychotherapy or on a waiting list				
**Completer**				
*3 months (t*_*2*_*)*	All participants	135	69	66
	Participants with TSP	106 (78.5)	59 (85.5)	47 (71.2)
*9 months (t*_*3*_*)*	All participants	112	58	54
	Participants with TSP	81 (72.3)	48 (82.8)	33 (61.1)
**ITT: best case**				
*3 months (t*_*2*_*)*	All participants	199	99	100
	Participants with TSP	135 (67.8)	88 (88.9)	47 (47.0)
*9 months (t*_*3*_*)*	All participants (n)	199	99	100
	Participants with TSP	119 (59.8)	86 (86.9)	33 (33.0)
**ITT: worst case**				
*3 months (t*_*2*_*)*	All participants	199	99	100
	Participants with TSP	139 (69.8)	59 (59.6)	80 (80.0)
*9 months (t*_*3*_*)*	All participants	199	99	100
	Participants with TSP	125 (62.8)	48 (48.5)	77 (77.0)

Results are expressed as absolute frequencies with percentages in parentheses.

In the completer analysis at t_2_, n = 115 patients were included in the regression model. Patients in the ACM group were significantly more likely to be in outpatient psychotherapy or on a waiting list compared to patients in the UC group at t_2_ (OR: 3.00 [1.12–8.07]; *p* = .029). At t_3_, n = 93 patients were included. Patients receiving ACM were more likely to be in outpatient psychotherapy or on a waiting list than patients in the UC group at t_3_ (OR: 4.78 [1.55–14.74]; *p* = .006).

In the ITT ‘best case’ analysis, n = 164 patients were included at both time points. Patients in the ACM group were more likely to be in outpatient psychotherapy or on a waiting list than the patients receiving UC at t_2_ (OR: 12.09 [4.78–30.60]; *p* = .000) and at t_3_ (OR: 17.72 [7.38–42.52]; *p* = .000).

In the ITT ‘worst case’ analysis, patients in the UC group were more likely to be in outpatient psychotherapy or on a waiting list compared to patients receiving ACM at t_2_ (OR: 0.42 [0.21–0.85]; *p* = .016) and at t_3_ (OR: 0.29 [0.15–0.59]; *p* = .001).

## Discussion

This study aimed to evaluate the effectiveness of aftercare case management by phone for patients with depression following inpatient treatment. Our results are consistent with previous research, which found mixed effects of case management programmes on symptom severity, quality of life and health care utilization [18, 28–31]. Regarding symptom severity, health-related quality of life and self-efficacy, patients in the ACM group did not differ significantly from those in the UC group. A possible explanation for the lack of statistical significance might be that the true effect was smaller than expected. Furthermore, most of the studies showing significant clinical benefits of case management were conducted in a primary care setting [21–25]. Patients in the current study had already received intensive psychotherapeutic treatment during their inpatient treatment prior to the intervention, whereas patients recruited from a primary care setting might not have received any previous psychotherapeutic treatment. Another explanation for the lack of effects could be the potentially low dose and duration of the intervention (six phone contacts every two weeks, with a mean duration of 22.7 minutes (SD = 7.1) each). However, studies that specifically investigate the dose-response relationship are lacking [20]. In contrast to psychotherapeutic interventions, the ACM provided in our study did not focus on disorder-specific complaints but on more general aspects such as motivating and providing consultation for the patients to coordinate their own aftercare; this might have also been a reason for the lack of effects on symptom severity. We assumed that the therapists in the inpatient units could use their existing therapeutic relationship and knowledge of the individual patient and were thus best qualified to ensure the continuity of treatment. However, it is conceivable that the patients expected a continuation of familiar psychotherapeutic support and might therefore have been disappointed with the ACM, which could have had a negative impact on the therapeutic relationship. According to a systematic review of 55 RCTs investigating different models of depression care, the monitoring and delivery of treatment was most effective when implemented by health professionals with a mental health background or by practice nurses [21]. The question of whether ACM is more effective when delivered by therapists from inpatients units or by other internal or external occupational groups should be investigated in future studies.

However, patients in the ACM group were more likely to be in outpatient psychotherapy or on a waiting list than patients in the UC group at t_2_ as well as at t_3_ (completer analyses). Treatment barriers to the clinical pathways of patients with depression, especially the long waiting periods for outpatient psychotherapy, are a common problem not only in the German health care system [7, 8, 10, 11] but also in other European countries and the United states [41, 42]. Therefore, a main purpose of the investigated intervention was to ensure continuity of care by providing improved follow-up regarding access to outpatient psychotherapy. Our results are consistent with those of a study investigating the effectiveness of telephone case management for Medicaid beneficiaries with depression [28], in which the intervention did not successfully reduce the average severity of depression but was effective in enrolling participants in mental health services. In Germany, a significant number of psychotherapeutic units are located at a distance from patients’ residence. Therefore, the telephone-based approach to managing patients after discharge from an inpatient unit seems to be particularly reasonable for overcoming long distances.

Due to the RCT design, it can be assumed that the internal validity of the current study was high. In this study sample, it is worth noting that the patients showed a relatively high symptom severity at the end of inpatient treatment (t_1_), which was, on average, in the range of moderate depression according to the BDI. This relatively high impairment at the end of inpatient treatment was unexpected and underlines the need to provide outpatient aftercare. To increase the external validity and support valid transferability to real-life health care, patients were recruited consecutively from four different care units after being screened for the inclusion criteria.

### Limitations

Even though we conducted drop-out analyses for t_2_ and t_3_ to identify potential sample biases, which did not yield any baseline differences between the completers and the non-completers the drop-out rates of 30% at t_2_ and 42% at t_3_ represent a limitation of the study. Due to changes in the study design and the primary statistical analysis, the technique used for calculation of the sample size does not perfectly fit to the analytic strategy used. Thus, we cannot be sure whether the power of the study was adequate, too low, or even too high. Furthermore we assumed that there are no systematic differences regarding the access to or kind of health care services. We are aware that this cannot be completely ruled out but unfortunately we did not collect data regarding patients’ residence. Another limitation that needs to be mentioned is the exclusion of patients with anxiety due to recruitment difficulties. To ensure a largely standardized procedure and therefore improve treatment adherence, the therapists were trained in their role as a coordinator and received a detailed manual. However, we are aware that the treatment adherence might be limited because the therapists’ procedures were not completely controlled. Further studies should use, e.g., audio recordings and perform content analyses of these recordings to control for treatment adherence.

## Conclusion

Although telephone-based ACM did not significantly improve symptom severity in patients with depressive disorders, we did find an effect of the intervention on the proportion of patients who managed to initiate outpatient psychotherapy. Thus, this newly developed aftercare intervention might be a valuable approach for overcoming the treatment barriers to patients’ clinical pathways. Further studies considering the limitations described above are needed to investigate the effectiveness of this type of aftercare intervention. Long-term analyses should address whether the short-term costs of aftercare will be exceeded by the possible savings, e.g., due to reduced hospitalizations and increased employment rates.

## Supporting information

S1 Table Changes in the study protocol. (DOCX)

S2 Table Results of ANCOVA analyses based on the ITT data regarding the primary and secondary outcomes (n = 199). Results are expressed as M (SD) = mean (standard deviation) and M (SE) = mean (standard error).
(DOCX)

S3 Table Results of ANCOVA analyses based on the completer data regarding the primary and secondary outcomes (n = 104). Results are expressed as M (SD) = mean (standard deviation) and M (SE) = mean (standard error).
(DOCX)

S1 Data set (XLSX)

S1 Study Protocol (PDF)

S1 Checklist (DOC)

S1 Clinical trial protocol ethics committee (DOC)

S1 Translation relevant parts clinical trial protocol (DOCX)
