# Supplementary material for: Effectiveness of telephone-based aftercare case management for adult patients with unipolar depression compared to usual care: A randomized controlled trial
Source: PLoS One. 2017 Oct 27;12(10):e0186967. doi: 10.1371/journal.pone.0186967 (PMC5659793; doi:10.1371/journal.pone.0186967)
Supplement: S1 Table — (DOCX) [file pone.0186967.s001.docx]

**S1 Table.** Changes in the Study Protocol

| **Domain** | **Original study  Protocol** | **Implemented change** | **Rationale/ Comments** |
| --- | --- | --- | --- |
| Patient population | Patients with depressive and anxiety disorders | Patients with depressive disorders | The recruitment of patients with anxiety disorders proved difficult, which led to a very low number of patients with anxiety disorders. Therefore, we decided to focus solely on the group of depressive patients. The required sample size was still sufficient for the effectiveness analyses. |
| Outcomes | Depressive symptom severity (BDI) as primary outcome, anxiety symptom severity as an additional outcome, assessed by Beck’s Anxiety Inventory (BAI). Further anxiety-specific measurements: Generalized Anxiety Disorder Scale (GAD-7), Body Sensations Questionnaire (BSQ), Agoraphobic Cognitions Questionnaire (ACQ), Mobility Inventory (MI), Social Phobia Scale (SPS), Social Interaction Anxiety Scale (SIAS) | Depressive symptom severity (BDI) as primary outcome. Anxiety symptom severity was removed as an additional outcome | The anxiety-specific measures were excluded from the analyses as the study focussed solely on the group of depressive patients. |
| Primary analyses | Analysis of covariance (ANCOVA) | Mixed model analyses with repeated measurements | The implemented model can be considered a generalized form of the planned analysis, allowing for using the data more efficiently and gaining more information on the effects of interest. Also, we preferred the mixed model approach because of its advantages in dealing with missing values. The ANCOVA analyses are reported in S2. |
