# Supplementary material for: Effectiveness of telephone-based aftercare case management for adult patients with unipolar depression compared to usual care: A randomized controlled trial
Source: PLoS One. 2017 Oct 27;12(10):e0186967. doi: 10.1371/journal.pone.0186967 (PMC5659793; doi:10.1371/journal.pone.0186967)
Supplement: S2 Table — Results are expressed as M (SD) = mean (standard deviation) and M (SE) = mean (standard error). (DOCX) [file pone.0186967.s002.docx]

**Sensitivity analysis of the primary and secondary outcomes using analysis of covariance**

S2 Table illustrates the results of the ANCOVA analyses based on the ITT data. Missing data were imputed using the Expectation-Maximization (EM) Algorithm.

Regarding the primary outcome of depressive symptom severity, the groups did not significantly differ at t_2_ (F_1, 122_= 3.145; *p*= .079; η2=.025) or at t_3_ (F_1,102_= 1.843; *p*=.178; η2=.018). With respect to the SF-8 and the EQ-5D, patients receiving ACM and those in the UC group did not show statistically significant differences. Regarding the SWE, the groups did not differ significantly at t_2_, whereas at t_3_ a significant difference was found.

**S2 Table.** Results of ANCOVA analyses based on the ITT data regarding the primary and secondary outcomes (n=199)

| **Outcome** | **Means** | | | | **Estimated mean difference (95% CI)** | ***p*** | **ES Eta-squared (η2)** |
| --- | --- | --- | --- | --- | --- | --- | --- |
|  | **Observed** | | **Estimated** | |  |  |  |
|  | M (SD) | | M (SE) | |  |  |  |
| Whole sample | **ACM** | **UC** | **ACM** | **UC** |  |  |  |
| **Primary Outcome** | | | | |  |  |  |
| **BDI-II** | | | | |  |  |  |
| *Baseline (t_1_)* | 23.5 (13.5) | 22.6 (12.9) |  |  |  |  |  |
| *3 months (t_2_), n=129* | 22.3 (13.1) n=61 | 24.6 (14.1) n=68 | 22.0 (1.5) | 25.2 (1.4) | -3.19 (-0.37 to 6.75) | .079 | .025 |
| *9 months (t_3_), n=109* | 19.6 (13.2) n=53 | 21.5 (12.0) n=56 | 19.6 (1.5) | 22.2 (1.5) | -2.61 (-1.20 to 6.43) | .178 | .018 |
| **Secondary Outcomes** | | | | |  |  |  |
| **SF-8** | | | | |  |  |  |
| *Baseline (t_1_)* | 37.1 (12.0) | 35.6 (11.2) |  |  |  |  |  |
| *3 months (t_2_), n=129* | 37.3 (11.1) n=63 | 35.2 (11.4) n=66 | 38.4 (1.5) | 35.8 (1.5) | 2.64 (-6.24 to 0.97) | .151 | .017 |
| *9 months (t_3_), n=108* | 38.7 (10.9)n=53 | 36.7 (10.9) n=55 | 40.0 (1.6) | 37.9 (1.6) | 2.02 (-6.17 to 2.13) | .337 | .009 |
| **EQ-5D** | | | | |  |  |  |
| *Baseline (t_1_)* | 0.61 (0.23) | 0.64 (0.21) |  |  |  |  |  |
| *3 months (t_2_), n=129* | 0.63 (0.22) n=63 | 0.63 (0.22) n=66 | 0.66 (0.02) | 0.63 (0.02) | 0.03 (-0.09 to 0.03) | .363 | .007 |
| *9 months (t_3_), n=108* | 0.64 (0.23) n= 54 | 0.66 (0.17) n=54 | 0.66 (0.03) | 0.66 (0.03) | -0.004 (-0.07 to 0.08) | .905 | .000 |
| **SWE** | | | | |  |  |  |
| *Baseline (t_1_)* | 12.8 (6.51) | 12.7 (6.96) |  |  |  |  |  |
| *3 months (t_2_), n=131* | 14.5 (6.8) n=63 | 12.9 (7.2) n=68 | 14.0 (0.7) | 12.5 (0.7) | 1.51 (-3.22 to 0.19) | .08 | .024 |
| *9 months (t_3_), n=110* | 15.3 (6.3) n=54 | 14.2 (7.2) n=56 | 15.5 (0.8) | 13.4 (0.8) | 2.10 (-4.10 to -0.13) | .037* | .042 |

Results are expressed as M (SD) = mean (standard deviation) and M (SE) = mean (standard error)
