# Supplementary material for: Effectiveness of telephone-based aftercare case management for adult patients with unipolar depression compared to usual care: A randomized controlled trial
Source: PLoS One. 2017 Oct 27;12(10):e0186967. doi: 10.1371/journal.pone.0186967 (PMC5659793; doi:10.1371/journal.pone.0186967)
Supplement: S3 Table — Results are expressed as M (SD) = mean (standard deviation) and M (SE) = mean (standard error). (DOCX) [file pone.0186967.s003.docx]

**Sensitivity analysis of the primary and secondary outcomes using analysis of covariance**

S3 Table illustrates the results of the ANCOVA analyses based on the Completer data.

Regarding the primary outcome of depressive symptom severity, the groups did not significantly differ at t_2_ (F_1, 93_= 3.745; *p*= .056; η2=.039) or at t_3_ (F_1, 93_= 1.863; *p*=.176; η2=.020). With respect to the SF-8 and the EQ-5D, patients receiving ACM and those in the UC group did not show statistically significant differences. Regarding the SWE, the groups differed significantly at t_2_ and at t_3_.

**S3 Table.** Results of ANCOVA analyses based on the Completer data regarding the primary and secondary outcomes (n=104)

| **Outcome** | **Means** | | | | **Estimated mean difference (95% CI)** | ***p*** | **ES Eta-squared (η2)** |
| --- | --- | --- | --- | --- | --- | --- | --- |
|  | **Observed** | | **Estimated** | |  |  |  |
|  | M (SD) | | M (SE) | |  |  |  |
| Whole sample | **ACM** | **UC** | **ACM** | **UC** |  |  |  |
| **Primary Outcome** | | | | |  |  |  |
| **BDI-II** | | | | |  |  |  |
| *Baseline (t_1_)* | 21.7 (13.5) | 21.3 (12.7) |  |  |  |  |  |
| *3 months (t_2_), n=100* | 20.5 (12.6) n=47 | 23.5 (13.1) n=53 | 20.3 (1.5) | 23.9 (1.5) | -3.63 (-0.10 to 7.35) | .056 | .039 |
| *9 months (t_3_), n=100* | 19.4 (12.8) n=47 | 21.4 (12.0) n=53 | 19.0 (1.5) | 21.4 (1.4) | -2.54 (-1.15 to 6.22) | .176 | .020 |
| **Secondary Outcomes** | | | | |  |  |  |
| **SF-8** | | | | |  |  |  |
| *Baseline (t_1_)* | 38.4 (12.2) | 36.6 (10.6) |  |  |  |  |  |
| *3 months (t_2_), n=100* | 38.1 (11.0) n=48 | 35.3 (11.2) n=52 | 38.7 (1.6) | 35.7 (1.6) | 2.98 (-7.12 to 1.16) | .156 | .022 |
| *9 months (t_3_), n=99* | 38.8 (11.1) n=47 | 37.0 (10.9) n=52 | 40.7 (1.7) | 38.7 (1.6) | 1.91 (-6.11 to 2.28) | .367 | .009 |
| **EQ-5D** | | | | |  |  |  |
| *Baseline (t_1_)* | 0.64 (0.21) | 0.64 (0.19) |  |  |  |  |  |
| *3 months (t_2_), n=99* | 0.67 (0.21) n=48 | 0.65 (0.21) n=51 | 0.68 (0.03) | 0.65 (0.03) | 0.03 (-0.09 to 0.04) | .369 | .009 |
| *9 months (t_3_), n=99* | 0.63 (0.25) n= 48 | 0.67 (0.18) n=51 | 0.65 (0.03) | 0.68 (0.03) | -0.03 (-0.05 to 0.10) | .499 | .005 |
| **SWE** | | | | |  |  |  |
| *Baseline (t_1_)* | 12.6 (6.58) | 13.9 (6.95) |  |  |  |  |  |
| *3 months (t_2_), n=101* | 15.2 (6.6) n=48 | 13.9 (7.1) n=53 | 15.2 (0.7) | 13.1 (0.7) | 2.10 (-3.96 to -0.24) | .027* | .051 |
| *9 months (t_3_), n=101* | 15.5 (6.5) n=48 | 14.2 (7.1) n=53 | 15.9 (0.8) | 13.7 (0.8) | 2.13 (-4.12 to -0.14) | .036* | .046 |

Results are expressed as M (SD) = mean (standard deviation) and M (SE) = mean (standard error)
