# Supplementary material for: Effectiveness of telephone-based aftercare case management for adult patients with unipolar depression compared to usual care: A randomized controlled trial
Source: PLoS One. 2017 Oct 27;12(10):e0186967. doi: 10.1371/journal.pone.0186967 (PMC5659793; doi:10.1371/journal.pone.0186967)
Supplement: S1 Study Protocol — (PDF) [file pone.0186967.s005.pdf]

Kivelitz et al. BMC Psychiatry (2015) 15:90
DOI 10.1186/s12888-015-0469-y

BMC Psychiatry

STUDY PROTOCOL Open Access

Effectiveness of case management-based aftercare coordination by phone for patients with depressive and anxiety disorders: study protocol for a randomized controlled trial

Laura Kivelitz[1*], Holger Schulz[1], Hanne Melchior[1†] and Birgit Watzke[1,2†]

Abstract

Background: Depressive and anxiety disorders are highly prevalent, but only a small percentage (approximately 50%) of patients receive appropriate treatment. Relevant barriers include communication and coordination gaps between different providers that result from the lack of integration between different care-giving systems. Aftercare following inpatient treatment represents one of these gaps because systematic follow-up care does not exist. Case management-based aftercare coordination by phone might be a promising approach to overcoming this gap and improving long-term treatment outcomes. Case management is a patient-centered and situation-based approach comprising systematic tracking and support of patients by a case manager.

The aim of this study is to evaluate the effectiveness of aftercare coordination by phone for patients with depressive and anxiety disorders.

Methods/design: The effectiveness of aftercare coordination will be investigated in a prospective randomized controlled trial in four psychotherapeutic inpatient routine care units (St. Franziska-Stift Bad Kreuznach, MediClin Seepark Klinik Bad Bodenteich, Segeberger Kliniken Gruppe Bad Segeberg and Luisenklinik Bad Dürrheim). The patients receiving aftercare coordination (intervention group; IG) will be compared with those who receive treatment as usual (TAU control group; CG). Eligible patients will be required to have a diagnosis of an anxiety and/or depressive disorder and a recommendation for follow-up outpatient psychotherapy.

The aftercare coordination consists of six phone contacts at intervals of two weeks that are performed by therapists in the inpatient units. The patients will complete questionnaires at discharge (t_1), 3 months after discharge (i.e., at the end of the intervention (t_2)) and 9 months after discharge (t_3). The primary outcome will be change in symptom severity from t_1 to t_3, the secondary outcomes will be health-related quality of life and the proportion of patients who manage to begin outpatient psychotherapy by t_3.

Discussion: This study will determine whether case management-based aftercare coordination by phone is an adequate approach for overcoming treatment barriers in the clinical pathways of patients with depressive and anxiety disorders. If proven effective, an accessible supplementary treatment approach that will help to maintain and even improve long-term treatment outcomes will be made available for patients following inpatient treatment.

Trial registration: ClinicalTrials.gov: (NCT02044913).

Keywords: Randomized controlled trial, Aftercare, Case management, Phone-based, Depression, Anxiety, Evaluation, Effectiveness

* Correspondence: l.kivelitz@uke.de
†Equal contributors
[1]Department of Medical Psychology, University Medical Center Hamburg-Eppendorf, Martinistraße 52, 20246 Hamburg, Germany
Full list of author information is available at the end of the article

BioMed Central

© 2015 Kivelitz et al.; licensee BioMed Central. This is an Open Access article distributed under the terms of the Creative Commons Attribution License (http://creativecommons.org/licenses/by/4.0), which permits unrestricted use, distribution, and reproduction in any medium, provided the original work is properly credited. The Creative Commons Public Domain Dedication waiver (http://creativecommons.org/publicdomain/zero/1.0/) applies to the data made available in this article, unless otherwise stated.

Background

Depressive and anxiety disorders are among the most prevalent mental disorders and cause significant personal, social and economic burdens. Despite the high 12-month prevalence rates of 15.3% for anxiety and 7.7% for depression in Germany [1] only approximately half of these patients receive appropriate treatment [2]. This finding is indicative of undertreatment and the need for the optimization in the care of patients with anxiety and depression. Significant barriers in the patients' pathways include communication and coordination problems between different services or providers [3-6]. The aftercare following inpatient treatment represents one of these gaps. After inpatient treatment, follow-up outpatient psychotherapy is clinically indicated for the majority of patients with anxiety and depressive disorders [7]. Here, the aims are to consolidate the treatment outcomes and to minimize the so-called rebound-effect, i.e., the long-term reduction in positive treatment effects following inpatient treatment. Although there is evidence supporting the effectiveness of inpatient treatment, treatment effects often decrease after treatment. A portion of patients who exhibit improvements at termination relapse and continue to seek help from a variety of mental health providers [8]. Depressive disorders are particularly highly recurrent, and the risk of a further episode increases with each additional episode; thus, the prevention of relapse is extremely desirable [9]. It can be assumed that the therapeutic alliance represents a protective factor against rebound effect; numerous studies have found that the therapeutic alliance is relevant to the outcome of psychotherapy [10] and the prevention of relapse [11]. However, the high risk of relapse indicates that follow-up support, e.g., outpatient psychotherapy, is necessary for patients to maintain long-term treatment outcomes after returning to everyday life. In addition to outpatient psychotherapy and primary and specialist care, patients can also profit from low-intensity offerings such as self-help groups and counseling. Thus, several aftercare treatment options exist, but many patients do not access follow-up treatment. Treatment barriers that result from a lack of integration of the different steps in care can occur on the systemic level (e.g., long waiting times for outpatient psychotherapy) and on the individual level (e.g., insufficient patient awareness of available treatments) [6].

Case management-based aftercare coordination by phone might be a promising approach to overcoming the gap between inpatient treatment and aftercare. Case management is a patient-centered and situation-based approach comprised of systematic tracking and support of patients by a case manager. The primary goal is to coordinate and integrate services across treatment settings by providing self-managing support and follow-up for patients [3].

Previous international research on the effectiveness of case management has consistently reported positive effects on treatment outcomes, e.g., symptoms, quality of life and patient satisfaction [12-14]. However, in Germany, case management programs for anxiety and depression have not yet been implemented. Because the German health-care system differs significantly from the systems of other countries, the transferability of results from international studies still requires to be verified [14]. Furthermore, the various case management-based approaches differ concerning their content and the health care setting in which they are implemented. The first effectiveness study of a case management model for patients with depression in the German health care system reported that phone-based case management in primary health care results in better treatment outcomes compared to regular care in terms of symptom reduction, medication adherence and patient satisfaction [15]. Here, the intervention focused on monitoring depression symptoms, adherence to medication and encouraging the patients to follow self-managing activities and engage in pleasant or social activities. In this study, the focus of the intervention will be on the coordination of an adequate aftercare for patients with anxiety and depressive disorders.

Objectives

This study aims first to evaluate the effectiveness of aftercare coordination by phone following inpatient treatment for patients with anxiety and depression. The primary outcome will be change in symptom severity from t_1 to t_3, and the secondary outcomes will be health-related quality of life, self-efficacy and the proportion of patients who managed to begin outpatient psychotherapy at follow-up (6 months after the intervention). Whether the patients who receive the aftercare coordination (i.e., the intervention group; IG) exhibit better treatment outcomes at the terminations of the intervention and follow-up compared to the patients who receive treatment as usual (TAU, i.e., the control group; CG) will be examined. Second, whether patient characteristics can predict the outcomes of aftercare coordination will be examined. Third, whether the therapeutic alliance during inpatient treatment has moderating effects on the primary and secondary outcomes will be examined. Forth, different anxiety-specific measures will be assessed to determine the changes in the symptom severities in patients with different anxiety disorders. Fifth, process evaluation will be conducted to identify the main contents of the aftercare coordination and to determine whether the patients benefit during the course of the intervention from the perspective of the therapists. Finally, the patients' satisfaction with the case management procedures and acceptance of the intervention will also be assessed.

Ethical approval
The study has been approved by the responsible local Ethics Committee of the Chamber of Physicians in Hamburg (Ref. Nr. PV4004) and will be conducted according to the principles of the Declaration of Helsinki (2013 version).

Methods/design
Study design
The study will be performed as prospective multicenter randomized controlled trial (Figure 1). The patients who receive aftercare coordination by phone (IG) will be compared to those who receive TAU without aftercare coordination by phone (CG). Measurements will be performed at three time points in each group. Baseline measures will be taken at discharge from the inpatient treatment (which will be the beginning of the intervention; t_1), and two follow-up measures will be taken after 3 months at the end of the intervention (t_2) and again 6 months after the end of the intervention (which will be 9 months after discharge from inpatient treatment; t_3).

Trial inclusion and exclusion criteria
The included patients will be required to be at least 18 years old and have a diagnosis of anxiety (F40.x, F41.x, according to ICD-10) [16] and/or depressive disorder (F32.x, F33.x, F34.1). The diagnoses will be validated with the Mini-DIPS diagnostic interview [17], which is a short version of the DIPS diagnostic interview for mental disorders [18]. Comorbid mental disorders or somatic comorbidity will not be an exclusion criterion.

Patients who are already in a concurrent outpatient psychotherapeutic treatment at admission that will be continued after the inpatient treatment will be excluded from the study because aftercare coordination will not be necessary for those patients. Further exclusion criteria include acute risk of suicide, acute psychosis or psychotic symptoms, insufficient German language skills and an inpatient treatment duration of less than three days.

Recruitment and data collection
The patients who fulfill the study inclusion criteria will be recruited consecutively by their therapists at the beginning of their inpatient treatment in the four cooperating psychotherapeutic inpatient units (St. Franziska-Stift Bad Kreuznach, MediClin Seepark Klinik Bad Bodenteich, Segeberger Kliniken Gruppe Bad Segeberg and Luisenklinik Bad Dürrheim). Prior to participation, the patients will be informed with oral and written information regarding the study by their therapists. After written informed consent is obtained, the patients will be randomly assigned to the IG or the CG.

Randomization
The patients will be randomized using a stratified block randomization with randomly varying block size to ensure concealment and equal group sizes, and the randomization will be stratified by the participating clinical units. The randomization will take place at the individual level at the University Medical Center Hamburg-Eppendorf one week before the beginning of the intervention. The allocation schedule was created with a computer program (Microsoft Excel, Windows) by a researcher at the University Medical Center Hamburg-Eppendorf. The therapists will be informed about the randomization outcome prior to the last psychotherapeutic session during inpatient treatment so that they can inform the patients about their group assignment before discharge.

Description of the intervention and control conditions
Intervention condition
The aftercare coordination is based on the concept of case management, which is a patient-centered approach that aims to support the patient in finding and organizing his or her individual aftercare treatment. After the inpatient treatment, the patients in the intervention group will receive six aftercare coordination phone contacts at intervals of two weeks that will be performed by their inpatient treatment therapists for 12 weeks. The task of the therapists will be to accompany and guide the patients in matters related to making plans and generating goals. The patients should

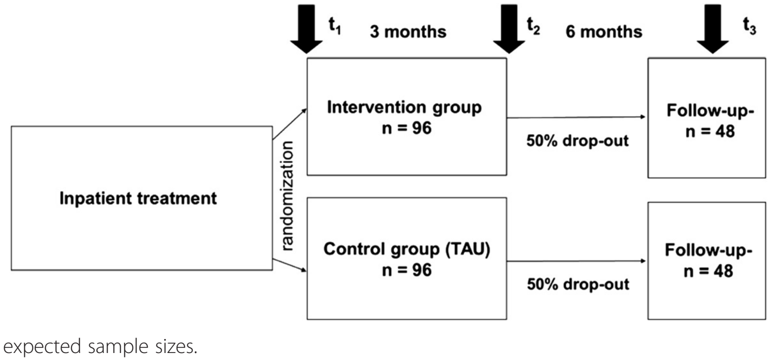

Figure 1 Study design and expected sample sizes.

be motivated and encouraged in terms of empowerment to become active in organizing their own aftercare treatment, and the therapists will provide feedback by monitoring the steps taken toward goal achievement. During the aftercare coordination, and in contrast to therapeutic interventions, the treatment of the patients' disorder-specific complaints will not be the primarily focus. The main contents of the phone contacts will be the patients' needs and problems associated with their aftercare coordination and supportive consultation from the therapist.

Prior to initiating the aftercare coordination, the therapists will be trained in their role as a coordinator and receive an elaborate manual that will provide guidelines for the phone contacts. This detailed manual contains helpful instructions for the therapist but allows sufficient freedom to tailor the coordination to the patients' needs, individual situations and conditions. The contents of the manual include descriptions of the aims of the aftercare coordination, the processes of the study and the phone contacts and instructions for dealing with specific situations that are demonstrated via examples. The phone contacts should last 20 to 30 minutes each. After each phone contact, the therapists will complete a self-developed questionnaire that includes process documentation of the duration and contents of the contact (see process measurements).

Control condition

After inpatient treatment, the patients in the CG will receive treatment as usual with routine care that does not involve the aftercare coordination by phone that the patients in the intervention group will receive. The patients in the control group will not have any contact with their therapists from the clinic after the inpatient treatment.

Outcome assessment

Primary outcome measures

The primary outcome measure is symptom severity, which will be assessed using Beck's Depression Inventory (BDI-II) [19]. The BDI-II consists of 21 items that are rated on a four-point Likert scale and yields a total score via the summation of the ratings for the individual items (range: 0 to 63; higher scores indicate higher symptom severity). The mean BDI cut-off scores for minimal, mild, moderate and severe depressive symptoms are 7.7, 19.1, 27.4 and 33.0, respectively. The BDI-II is a reliable and valid measure of depression symptoms with an internal consistency ranging from .92 to .93. This instrument has been widely used and is sensitive to change.

Secondary outcome measures

The secondary outcomes include health-related quality of life as measured with the EuroQol-5D [20] and the Short Form 8 Health Survey (SF8) [21]. The >EuroQol-5D is a generic instrument that measures health-related quality of life in five dimensions; i.e., mobility, self-care, usual activities, pain/discomfort and anxiety/depression. Health-related quality of life will also be assessed with a visual analogue scale (range: 0 to 100; higher ratings indicate higher quality of life). The SF-8 is a short version of the SF-36 [22] allows for the calculation of scores for physical and mental health (range: 0 to 100; higher scores indicate better statuses). This instrument has good psychometric properties with an internal consistency between .70 and .88 [23].

As an additional secondary outcome, the proportion of patients who managed to organize outpatient psychotherapy at follow-up will be assessed with self-report items. Whether self-efficacy can be improved by the case management-based intervention that aims to empowering the patients to become active will also be investigated. The German General Self-Efficacy Scale (SWE) [24] will be used to assess self-efficacy. This scale comprises 10 four-point Likert-scale items that range from 1 ('totally disagree') to 4 ('totally agree'). The questionnaire includes items such as "If there are challenges, I can find a way to succeed" and "I can find a solution for every problem". The summed scores range between 10 and 40, and higher scores indicate greater general self-efficacy. The internal consistency of this instrument ranges from .80 to .90 [24].

Additional measures

To investigate whether patient characteristics can predict the outcomes of aftercare coordination, the following demographic and clinical characteristics will be collected via a self-report questionnaire: age, gender, marital status and partnership, children, level of education, employment status and occupational situation, and medical and psychosocial treatment prior to the inpatient treatment. To examine whether the therapeutic alliance is associated with the primary outcome, the therapeutic relationship will be measured with the Helping Alliance Questionnaire (HAQ-S) [25], which consists of 11 items that are rated on a six-point Likert scale ranging from −3 ('strongly disagree') to 3 ('strongly agree'). A total score is calculated, and higher total scores are indicative of better alliances. Research has demonstrated the good reliability and validity of this instrument [26].

Anxiety-specific measures

Additionally, Beck's Anxiety Inventory (BAI) [27] will be utilized to measure the changes in anxiety symptom severity. The BAI contains 21 items that assess the degree to which the respondent has been affected by physical or cognitive symptoms of anxiety during the past week. The BAI items are also meant to reflect panic attack symptoms. The total score ranges from 0 to 63, and high scores indicate more severe anxiety. The internal consistency ranges from .83 to .95. To gather data regarding changes in the severity

of the symptoms of specific anxiety disorders, the following diagnosis-specific outcome measures will be used only in the questionnaires for the patients with anxiety diagnoses. Generalized anxiety disorder symptoms will be assessed with the seven-item Generalized Anxiety Disorder Scale (GAD-7) [28], which measures the symptom severity over the previous two weeks on a four-point Likert scale that ranges from 0 (“not at all”) to 3 (“nearly every day”). The sum score ranges from 0 to 21. Scores of 5, 10, and 15 represent the cut-off points for mild, moderate and severe anxiety, respectively. This instrument is a reliable and valid measure of anxiety with an internal consistency of .89 [29]. Panic disorder symptoms will be measured with the Body Sensations Questionnaire (BSQ) and the Agoraphobic Cognitions Questionnaire (ACQ) [30]. The BSQ contains 17 items concerning the degree to which the patient fear somatic symptoms that are commonly associated with anxiety and panic attacks. This inventory assesses the fear of certain body sensations. The items are rated on a five-point Likert scale ranging from 1 = “not frightened or worried by this sensation” to 5 = “extremely frightened by this sensation”. The total score is calculated by averaging the individual item ratings. The internal consistencies in different samples range from .80 to .95 [31]. The ACQ measures the frequency of fear-related cognitions, and the fear of negative social or health consequences of fear. The ACQ contains 14 items (6 behavioral-social and 8 physical items) that are rated on a five-point Likert scale ranging from 1 = “the thought never occurs” to 5 = “the thought always occurs when I am nervous”. The internal consistency in different samples ranges from .74 to .87 [31]. The total score is derived by averaging the individual item ratings.

Agoraphobic symptoms will be assessed with the Mobility Inventory (MI) [32], which is a 26-item self-report questionnaire that was designed to assess the severity of the avoidance of common agoraphobic situations. Each situation is rated on a five-point Likert scale ranging from 0 (never avoids) to 4 (always avoids). Each situation is rated twice to reflect the degrees to which each situation is avoided by the agoraphobic participant when he is alone and when he is accompanied. The MI is scored by calculating the average avoidance rating across all situations for the Avoidance Alone and Avoidance Accompanied Scales. The internal consistency in different samples ranges from .85 to .97 [31].

The Social Phobia Scale (SPS) and the Social Interaction Anxiety Scale (SIAS) [33] will be used to measure social anxiety. The SPS assesses anxiety in 20 performance situations, and the SIAS was constructed to measure anxiety in 20 social interaction situations. Each situation is rated on a 4-point scale ranging from 0 to 4. The sum scores for both questionnaires range from 0 to 80, and higher scores represent greater anxiety about being observed by others or social interactional anxiety. The cut-offs are >24 for the SPS and >35 for the SIAS. The internal consistencies in different German samples range from .94 to .95 for the SPS and .86 to .88 for the SIAS [34].

Process measurements

To identify the main contents and to determine if the patients benefit during the course of the aftercare coordination from the perspective of the therapists, a process evaluation will be conducted. After every phone contact, the therapist will answer questions such as ‘Which steps have been generated, and did the patient carry out these steps?’, ‘Is the patient motivated and open to the aftercare coordination and benefiting from it?’ and ‘What was the central task of the therapist during the phone contact, e.g., motivating the patient, informing and consulting, planning the next steps, and controlling and monitoring of the patient’s progress?’ in the developed questionnaire.

Furthermore the patients’ satisfaction with the intervention procedures and acceptance of the aftercare coordination will be assessed with 25 self-generated items. For example, the items will include “The phone contacts helped me in finding my way back to everyday life” and “My therapist and I always discussed the individual steps of my aftercare support” and will be rated on a five-point Likert scale ranging from 1 (‘totally agree’) to 5 (‘totally disagree’). Additionally, the patients will be asked to answer some open questions such as “What could be improved regarding the aftercare coordination?”.

Statistical analyses

The primary analysis will focus on the changes in symptom severity from t_1 to t_3 as assessed with the BDI-II. Comparisons between the IG and the CG will be calculated with mixed model analyses of covariance (ANCO VAs) with symptom severity as the dependent variable and gender and initial symptom severity as covariates. An advantage of this method is that it includes all available data even if the data from another time point for a specific person are missing. Other secondary analyses will be performed regarding changes in health-related quality of life, self-efficacy and anxiety symptom severity from t_1 to t_2 and from t_1 to t_3. Following the intention-to-treat approach (ITT), we will analyze all randomized participants in the primary analysis to avoid biases such as non-random attrition of the participants. Additionally, a sensitivity analysis following the per-protocol approach will be conducted. This analysis will be performed including only the participants that have completed all measurements. To investigate whether the patient characteristics (e.g., age, level of education, and psychosocial treatment before the inpatient treatment) and patient ratings of the therapeutic alliance predict the outcomes

of the aftercare coordination, different regression analysis models will be applied to the primary and secondary outcomes. Process evaluation and data about the patients' satisfaction and acceptance will be investigated via descriptive and qualitative analyses. We will also conduct drop-out analyses to compare the demographic and clinical baseline characteristics of the completers and non-completers (*t*- and χ^2-tests). The analyses will be conducted with SPSS 21.

Power calculation

We expect a moderate effect size, which is defined as f = 0.25 according to Cohen [35], for the primary outcome because recent studies that have investigated aftercare programs have reported moderate effect sizes in terms of changes in symptom severity (e.g., depressiveness) [36]. We expect a reduction of the error variance of approximately 30% due to the inclusion of initial symptom severity (BDI at t_1) as a covariate (which can be expected to be uncorrelated with the study conditions due to the randomization). These considerations lead to an adjusted effect size of f = 0.29. To detect a moderate effect between the intervention and control groups in terms of the symptom severity with a power of 80% and an α of 0.05, a sample size of N = 96 (48 per group) is required. Based on an expected dropout rate of approximately 50% from baseline (t_1) to 9 months of follow-up (t_3), we aim to include a sample of N = 192 (96 per group) to perform the per-protocol analysis with a sufficient sample size.

Discussion

This paper presents a study protocol for a prospective multicenter RCT evaluating the effectiveness of case management-based aftercare coordination by phone following inpatient treatment for patients with depressive and anxiety disorders. The evaluated intervention is a manualized aftercare approach that is delivered by specially trained therapists that focuses on supporting the patients in finding and organizing their individual aftercare treatment. Considering the positive effects of case management for patients with depressive and anxiety disorders in terms of symptom reduction and quality of life that have been reported in previous studies in other settings [12,13,15,14], we hypothesize that aftercare coordination following inpatient treatment will help to minimize the risk of relapse and consolidate and even improve long-term treatment outcomes. To the extent of our knowledge, the present RCT will be the first to examine the effects of case management-based aftercare coordination by phone for patients with depressive and anxiety disorder. Acquiring access to adequate follow-up treatment should help the patients to maintain positive treatment outcomes after returning to everyday life. Although initiating outpatient treatment is the decisive aim of the intervention, it is not the primary or only criterion of success. Depending on the structural circumstances in the health care system, there might be situations in which it is not possible to initiate an adequate aftercare treatment, e.g., in cases of long waiting times for outpatient psychotherapy [37], within the three month of the intervention. However, there are several potential aftercare treatment possibilities other than outpatient psychotherapy and primary and specialist care, including low-threshold offers, e.g., self-help groups and consulting centers, from which the patients can benefit. Therefore, the likelihood of initiating adequate aftercare should be reasonable. However, the main aim of the intervention is to provide the patients with tools that enable them to continue organizing their aftercare themselves. Presumably, in addition to symptom severity, health-related quality of life and self-efficacy will also be improved by the case management-based intervention; therefore, these latter parameters will also be included as secondary outcome measures. Learning to manage aftercare treatment successfully themselves might be an experience that promotes feelings of satisfaction and self-efficacy in the patients. As an additional secondary outcome, the proportion of patients who managed to begin outpatient psychotherapy at follow-up will be assessed. It is expected to be higher in the IG than in the CG due to the supporting aftercare intervention. Presumably, the study will have high external validity because the participants will be consecutively recruited from routine care and routine clinical settings. If the intervention is found to be effective and accepted by the patients and professionals, an accessible supplementary treatment approach that can help to overcome barriers in the clinical pathways will be made available for patients following inpatient treatment. Treatment barriers due to the lack of integration of the different caregiving systems are common problems in the German health care system [5]. Therefore, aftercare coordination could also be adapted to the needs of and applied to patients with other mental disorders and chronic diseases.

Competing interests

The authors declare that they have no competing interests.

Authors' contributions

BW and HS conceptualized the study and obtained funding. BW and HM are the primary investigators of the study and therefore are consistently mentoring the entire study processes and article preparation. LK organize the network, recruitment process and data collection and management. HM and BW shared senior authorship. All authors contributed, read and approved the final manuscript.

Authors' information

BW is the head of the Department of Clinical Psychology and Psychotherapy Research at the University of Zürich and a certified psychotherapist. HS is professor and research coordinator of the Department of Medical Psychology of the University Medical Center Hamburg-Eppendorf. HM is postdoctoral research group leader and LK PhD student and research associate at the Department of Medical Psychology of the University Medical Center Hamburg-Eppendorf.

Acknowledgements
The study is funded by Germany's Federal Ministry of Education and Research and the German Federal Pension Fund as part of the research priority 'Chronic Diseases and Patient Orientation'. The project was competitively adopted based on a detailed research proposal (reference number: 0421-FSCP-Z202). We would like to thank Prof. Dr. Heinz Rüddel (St. Franziska-Stift Bad Kreuznach), Sabine Zahn (MediClin Seepark Klinik Bad Bodenteich) and Prof. Dr. Lutz Götzmann (Segeberger Kliniken Gruppe Bad Segeberg, Prof. Dr. Dr. Norbert Grulke (Luisenklinik Bad Dürrheim) for their cooperation.

Author details
[1]Department of Medical Psychology, University Medical Center Hamburg-Eppendorf, Martinistraße 52, 20246 Hamburg, Germany. [2]Clinical Psychology and Psychotherapy Research, Institute of Psychology, University of Zurich, Binzmühlestrasse 14/16, CH-8050 Zurich, Switzerland.

Received: 16 February 2015 Accepted: 8 April 2015
Published online: 22 April 2015

References

1. Jacobi F, Höfler M, Strehle J, Mack S, Gerscheler A, Scholl L, et al. Mental disorders in the general population - Study on the health of adults in Germany and the additional module mental health (DEGS1-MH). Nervenarzt. 2014;85:77–87.
2. Mack S, Jacobi F, Gerschler A, Strehle J, Höfler M, Busch MA, et al. Self-reported utilization of mental health services in the adult German population–evidence for unmet needs? Results of the DEGS1-Mental Health Module (DEGS1-MH). Int J Methods Psychiatr Res. 2014;23(3):289–303.
3. Klesse C, Bermejo I, Härter M. Innovative care models for treating depression. Nervenarzt. 2007;78:585–96.
4. Collins KA, Westra HA, Dozois DJA, Burns DD. Gaps in accessing treatment for anxiety and depression: challenges for the delivery of care. Clin Psychol Rev. 2004;24:583–616.
5. Schulz H, Barghaan D, Koch U, Harfst T. Die Versorgung von Patienten mit Psychischen Störungen. In: Wittchen H-U, Hoyer J, editors. Klinische Psychologie und Psychotherapie. Berlin: Springer; 2011. p. 361–80.
6. Kivelitz L, Watzke B, Schulz H, Härter M, Melchior H. Health care barriers on the pathways of patients with anxiety and depressive disorders – a qualitative interview study. Psychiat Prax. 2014. doi: 10.1055/s-0034-1370306.
7. Harfst T, Koch U, Schulz H. Treatment recommendations in psychosomatic rehabilitation - empirical analyses based on the pension insurers' standardized discharge report. Rehabilitation. 2002;41(6):407–14.
8. Lambert MJ. The Efficacy and Effectiveness of Psychotherapy. In: Lambert MJ, editor. Bergin and Garfield's Handbook of Psychotherapy and Behavior Change. 6th ed. Hoboken, New Jersey: John Wiley; 2013. p. 169–218.
9. Burcusa SL, Iacono WG. Risk for recurrence in depression. Clin Psychol Rev. 2007;27(8):959–85. doi:http://dx.doi.org/10.1016/j.cpr.2007.02.005.
10. Martin DJ, Garske JP, Davis MK. Relation of the therapeutic alliance with outcome and other variables: A meta-analytic review. J Consult Clin Psychol. 2000;68:438–50.
11. Weck F, Weigel M, Hautzinger M, Barocka A, Schlösser RG, Stangier U. Relapses in recurrent depression 1 year after psychoeducational treatment: The role of therapist adherence and competence, and the therapeutic alliance. Psychiatry Res. 2012;195(1):51–5.
12. Gensichen J, Beyer M, Muth C, Gerlach FM, Von Korff M, Ormel J. Case management to improve major depression in primary health care: a systematic review. Psychol Med. 2006;36(1):7–14.
13. Rollman BL, Belnap B, Mazumdar S, et al. A randomized trial to improve the quality of treatment for panic and generalized anxiety disorders in primary care. Arch Gen Psychiatry. 2005;62(12):1332–41.
14. Lehmann S, Domdey A, Bramesfeld A. Telephone Case Management: Is it Beneficial for the Care of Depression Patients in Germany? A Systematic Literature Survey. Gesundheitswesen. 2010;72(05):e33–7. doi:10.1055/s-0029-1242769.
15. Gensichen J, von Korff M, Peitz M, Muth C, Beyer M, Güthlin C, et al. Case management for depression by health care assistants in small primary care practices a cluster randomized trial. Ann Intern Med. 2009;151(6):369–78.
16. Dilling H, Mombour W, Schmidt MH. International classification of diseases, 10th rev. Bern: Huber; 1991.
17. Margraf J. Mini-DIPS Diagnostisches Kurz-Kurzinterview bei psychischen Störungen. Lehrbuch der Verhaltenstherapie. Berlin: Springer; 1994.
18. Schneider S, Margraf J. DIPS - Diagnostisches Interview bei psychischen Störungen. Berlin Heidelberg: Springer-Verlag; 2011.
19. Beck AT, Epstein N, Brown G. Manual of the Beck Depression Inventory-II. San Antonio, TX: Psychological Corporation; 1996.
20. The EuroQol Group. EuroQol - a new facility for the measurement of health-related quality of life. Health Policy. 1990;16(3):199–208.
21. Ellert U, Lampert T, Ravens-Sieberer U. Measuring health-related quality of life with the SF-8. Normal sample of the German population. Bundesgesundheitsbl. 2005;48:1330–7.
22. Ware JE. SF-36 Health Survey Update. Spine. 2000;25(24):3130–9.
23. Ware JE, Kosinski M, Dewey JE, Gandek B. How to score and interpret single-item health status measures: a manual for users of the of the SF-8 health survey. Lincoln, RI: QualityMetric, Incorporated; 2001.
24. Schwarzer R, Jerusalem M. Skalen zur Erfassung von Lehrer- und Schülermerkmalen. Dokumentation der psychometrischen Verfahren im Rahmen der Wissenschaftlichen Begleitung des Modellversuchs Selbstwirksame Schulen. Berlin: Freie Universität Berlin; 1999.
25. Luborsky L, Crits-Christoph P, Alexander L, Margoli M, Cohen M. Two helping alliance methods for predicting outcomes of psychotherapy: A counting signs vs. a global rating method. J Nerv Ment Dis. 1983;171:480–91.
26. Bassler M, Potratz B, Krauthauser H. The "Helping Alliance Questionnaire" (HAQ) by Luborsky. Psychotherapeut. 1995;40(1):23–32.
27. Beck AT, Epstein N, Brown G, Steer RA. An inventory for measuring clinical anxiety: psychometric properties. J Consult Clin Psychol. 1988;56:893–7.
28. Spitzer RL, Kroenke K, Williams JBW, Lowe B. A brief measure for assessing generalized anxiety disorder - The GAD-7. Arch Intern Med. 2006;166(10):1092–7.
29. Löwe B, Decker O, Müller S, Brähler E, Schellberg D, Herzog W, et al. Validation and Standardization of the Generalized Anxiety Disorder Screener (GAD-7) in the General Population. Med Care. 2008;46(3):266–74. doi:10.1097/MLR.0b013e318160d093.
30. Chambless DL, Caputo GC, Bright P, Gallagher R. Assessment of fear of fear in agoraphobics: the body sensations questionnaire and the agoraphobic cognitions questionnaire. J Consult Clin Psychol. 1984;52(6):1090–7.
31. Ehlers A, Margraf J, Chambless D. Fragebogen zu körperbezogenen Ängsten, Kognitionen und Vermeidung (2. überarb. u. Neunormierte Aufl.). Göttingen: Beltz; 2001.
32. Chambless DL, Caputo GC, Jasin SE, Gracely EJ, Williams C. The Mobility Inventory for Agoraphobia. Behav Res Ther. 1985;23:35–44.
33. Mattick RP, Clarke JC. Development and validation of measures of social phobia scrutiny fear and social interaction anxiety. Behav Res Ther. 1998;36(4):455–70.
34. Rabung S, Jaeger U, Streeck U, Leichsenring F. Psychometric analysis of the Social Phobia Scale (SPS) and the Social Interaction Anxiety Scale (SIAS) in an inpatient sample. Diagnostica. 2006;52(3):143–53.
35. Cohen J. Statistical power analysis for the behavioral sciences. Hillsdale, NJ, England: Lawrence Erlbaum Associates; 1988.
36. Deck R, Schramm S, Hüppe A. Supported Own Initiative of Rehabilitation Patients ("New Credo") – A Successful Model? Rehabilitation. 2012;51(05):316–25. doi:10.1055/s-0031-1291279.
37. Bundespsychotherapeutenkammer. BPtK-Studie zu Wartezeiten in der ambulanten psychotherapeutischen Versorgung. Umfrage der Landespsychotherapeutenkammern und der BPtK. 2011.

Submit your next manuscript to BioMed Central and take full advantage of:

- **Convenient online submission**
- **Thorough peer review**
- **No space constraints or color figure charges**
- **Immediate publication on acceptance**
- **Inclusion in PubMed, CAS, Scopus and Google Scholar**
- **Research which is freely available for redistribution**

Submit your manuscript at
www.biomedcentral.com/submit

BioMed Central
