# Supplementary material for: Effectiveness of telephone-based aftercare case management for adult patients with unipolar depression compared to usual care: A randomized controlled trial
Source: PLoS One. 2017 Oct 27;12(10):e0186967. doi: 10.1371/journal.pone.0186967 (PMC5659793; doi:10.1371/journal.pone.0186967)
Supplement: S1 Clinical trial protocol ethics committee — (DOC) [file pone.0186967.s007.doc]

**Ethik-Antrag**

**zur Vorlage bei der zuständigen Ethik-Kommission
der Ärztekammer Hamburg**

Grundsätze für die Arbeit der Ethik-Kommission (Stand: Juni 2002)

(in Anlehnung an die ‘Grundsätze für die ordnungsgemäße Durchführung der klinischen Prüfung von Arzneimitteln vom 9.12.87’ und die ‘GCP-Richtlinien vom 11.7.90’ in Verbindung mit §§ 40-42 AMG (für Arzneimittelstudien) sowie §§ 20-23 MPG (für Medizinproduktestudien).

Voraussetzung für eine Anfrage an die Ethik-Kommission zur Erlangung eines Primär-Votums (für den gemäß § 40 AMG oder § 20 MPG verantwortlichen Leiter der Klinischen Prüfung) ist die Übersendung kompletter Unterlagen:

• vollständig beantworteter Fragenkatalog,

• Prüfplan (Studienprotokoll),

• Patienten-/Probandenaufklärung und Einwilligungserklärung,

• Versicherungsnachweis,

• Investigator’s Brochure.

Handelt es sich um eine Studie, die nicht unter die Bestimmungen des AMG oder MPG fällt, gilt das Vorstehende analog. Die Ethik-Kommission bittet um eingehende Ausführungen zu den nachfolgend genannten Fragen (unter Angabe der jeweiligen Überschrift) ohne Verweise auf Angaben im Studienprotokoll. Nichtzutreffendes bitten wir mit ‘entfällt’ zu beantworten.

Bei Anfragen nach dem Medizinproduktegesetz (MPG) benötigt die Kommission Angaben zur sicherheitstechnischen Unbedenklichkeit des Medizinproduktes sowie Angaben darüber, ob die Studie mit einem CE-gekennzeichneten Medizinprodukt durchgeführt wird oder der Erlangung des CE-Kennzeichens dient.

Bitte übersenden Sie alle Unterlagen in dreifacher Ausfertigung (bei Prüfungen unter Anwendung von Strahlentherapie in vierfacher Ausfertigung)

1. Persönliche Angaben

### 1.1 Namen und Dienststellungen des Leiters der Klinischen Prüfung (LKP) (entspr. § 40 AMG oder § 20 MPG) und seiner Mitarbeiter

### LKP:

Birgit Watzke, PD Dr. phil., Dipl. Psych., Psychologische Psychotherapeutin

Institut und Poliklinik für Medizinische Psychologie, Zentrum für Psychosoziale Medizin, Universitätsklinikum Hamburg Eppendorf

Martinistraße 52 (Haus W26), 20246 Hamburg

Tel.: ++49 - (0)40 – 7410-54883 / Fax: -54940; E-Mail: [watzke@uke.uni-hamburg.de](mailto:watzke@uke.uni-hamburg.de)

Holger Schulz, Prof. Dr. phil., Dipl. Psych.,

Institut und Poliklinik für Medizinische Psychologie, Zentrum für Psychosoziale

Medizin, Universitätsklinikum Hamburg Eppendorf

Martinistr. 52 (Haus W26), 20246 Hamburg

Tel.: ++49- (0)40 - 7410- 56806/ Fax: -54940; E-Mail: schulz@uke.uni-hamburg.de

### Mitarbeiterin:

Hanne Melchior, Dr. phil., Dipl.-Psych.

Institut und Poliklinik für Medizinische Psychologie, Zentrum für Psychosoziale Medizin,

Universitätsklinikum Hamburg-Eppendorf, Martinistraße 52 (Haus W26), 20246 Hamburg

Tel.: ++49 - (0)40 - 7410-53871; E-Mail: h.melchior@uke.de

### 1.2 Nachweise (nur bei erstmaliger Anfrage erforderlich) über die wissenschaftliche Qualifikation des LKP entsprechend § 40 (1) 4. AMG; § 20 (1) 4. MPG.

Entfällt, da keine erstmalige Anfrage.

### 1.3 Finanzierung der Studie (Sponsor)?

Bundesministerium für Bildung und Forschung und Deutsche Rentenversicherung Bund im Rahmen des Forschungsförderschwerpunkts „Chronische Krankheiten und Patienten­orientierung“.

### 1.4 Anfragen an die Ethik-Kommission sind gemäß der Gebührenordnung der Ärztekammer Hamburg gebührenpflichtig:

### - An welche Person/Institution soll der Gebührenbescheid gerichtet werden?

Birgit Watzke, PD Dr. phil.

Institut und Poliklinik für Medizinische Psychologie, Zentrum für Psychosoziale Medizin, Universitätsklinikum Hamburg Eppendorf

Martinistraße 52 (Haus W26), 20246 Hamburg

2. Beschreibung und wissenschaftliche Begründung des Projektes,

Es soll in einem ersten Studienteil eine Ist-Analyse von Chronifizierungsprozessen und Behandlungswegen bei Patienten mit Angst- und depressiven Erkrankungen durchgeführt sowie, in einem zweiten Studienteil darauf aufbauend, die Effektivität einer telefonbasierten Nachsorge-Koordination (NK) nach stationärer Rehabilitation evaluiert werden. Hypothese: Die telefonbasierte NK führt zu besserem Behandlungsergebnis als Treatment as Usual (TAU; kein spezifisches Nachsorgeangebot).

### 2.1 Erläuterung des Versuchsziels

Mit dem beantragten Projekt wird in Studienteil 1 die Erstellung einer Ist-Analyse zur Situation von Patienten mit Angsterkrankung und von Patienten mit Depression sowie darauf aufbauend die Ableitung von Empfehlungen verfolgt, wobei folgende Hauptbereiche fokussiert werden:

(a) langfristiges Krankheitsgeschehen (Beginn & Verlauf) und Chronifizierungsprozesse

(b) langfristige Behandlungswege (‚Patientenkarrieren’) der Patienten durch die Institutionen des Gesundheitssystems; Steuermechanismen; Adäquatheit der Behandlungswege (Abgleich mit vorliegenden Leitlinien)

(c) Unterstützungs- und Behandlungsbedarfe; fördernde und hemmende Faktoren bei der Inanspruchnahme von Angeboten; Optimierungsbedarf hinsichtlich der Behandlungsangebote und Steuermechanismen.

Der Schwerpunkt der Analyse liegt dabei auf den unter (c) genannten Inhalten, da diese direkt handlungsleitend für eine mögliche Optimierung der Versorgung von Patienten mit chronischem Krankheitsverlauf sein können. Empfehlungen sollen dementsprechend insbesondere für die Verbesserung von Steuermechanismen und die Ausgestaltung von Unterstützungs- und Behandlungsangeboten abgeleitet werden. Hypothesen werden zu diesem explorativ ausgerichteten Studienteil nicht formuliert.

Studienteil 2 verfolgt die Evaluation einer Case-Management-orientierten Nachsorge-Koordination (NK) mittels Telefon für Patienten mit chronischer Depression bzw. chronischer Angsterkrankung nach stationärer Rehabilitation. Die Nachsorge-Koordination (NK) soll dabei den Übergang von der stationärer Rehabilitationsmaßnahme zu einer für den Patienten adäquaten Nachsorgemaßnahme sichern, d. h. es soll auf Systemebene den Behandlungspfad des Patienten nach Abschluss der stationären Rehabilitationsmaßnahme optimieren (ohne ein eigenes therapeutisches Nachsorgeangebot darzustellen). Während der strukturelle Rahmen der zu überprüfenden Intervention (NK) vorgegeben ist, orientiert sich die inhaltliche Ausgestaltung (was wird den Patienten an Interventionen angeboten?) an den Ergebnissen von Studienteil 1. Zielsetzungen der Evaluation umfassen die Überprüfung (A) der Effektivität, (B) der Akzeptanz sowie (C) der Patientenzufriedenheit.

Der Schwerpunkt der Evaluation bezieht sich auf die Effektivitätsüberprüfung mit folgender Hauptfragestellung: Kann eine telefonbasierte Nachsorge-Koordination die Effekte der Rehabilitation von Patienten mit chronischen Depressionen bzw. Angsterkrankungen sichern bzw. ausbauen?

Spezifisch ist dabei zu untersuchen:

(A.1.) Unterscheiden sich Patienten mit NK hinsichtlich ihres mittelfristigen Behandlungsergebnisses (d. h. zum 6-Monats-Follow-Up) von Patienten, die keine NK erhalten (d.h. Patienten der Regelversorgung bzw. mit ‚Treatment as usual’, TAU)? Das primäre Outcome stellt die gesund­heits­bezogene Lebensqualität dar. Sekundäre Outcomebereiche sind: Symptomatik, weitere krankheitsbezogene und sozialmedizinische Variablen (u.a. berufliche Reintegration, Inanspruchnahme von Behandlungen; Behandlungsspfade nach der Rehabilitation), Selbstwirksamkeitserwartung/ Empowerment sowie Medikamentencompliance. Obwohl auch kostenrelevantes Outcome überprüft wird, soll auf eine explizite Effizienzüberprüfung verzichtet werden: Da das Konzept einer Nachsorge-Koordination erst mittel- bis langfristige Einsparungen erwarten lässt, ist eine Erhebung im Rahmen des Förderzeitraumes nicht möglich und sollte darauf aufbauend erfolgen.

Weitere Fragestellungen:

(A.2) Für welche Patienten erweist sich NK als (besonders) effektiv? Lassen sich patientenseitige Prädiktoren für das Outcome identifizieren? Hierbei sollen vor allem gender- und diagnosespezifische Auswertungen vorgenommen werden, es sollen aber auch weitere Merkmale wie insbesondere die Ausgangsbelastung der Patienten, vorliegende Komorbidität sowie Alter und Bildung als potentielle Prädiktoren überprüft werden.

(A.3) Wie wird die Nachsorge-Koordination von Patienten und Therapeuten akzeptiert? Wie zufrieden sind Patienten und Therapeuten mit dem Angebot?

(A.4) Welche Prozessmerkmale (inhaltliche und strukturelle Merkmale der NK, z. B. besprochene Inhalte, Gesprächsstil, Länge und Dauer der Gespräche) lassen sich im Rahmen der Nachsorge-Koordination identifizieren?

Als Hypothese zur Hauptfragestellung A.1 wird erwartet, dass NK der TAU-Bedingung hinsichtlich des Behandlungs­erfolges überlegen ist. Dementsprechend wird erwartet, dass Patienten mit NK von Rehabilitations­beginn zu Follow Up sich hinsichtlich der definierten Zielbereiche (siehe 2.5) in einem signifikant höheren Ausmaß verbessern als Patienten mit TAU. Zu den Fragestellungen A.2, A.3 und A.4 werden aufgrund ihres explorativen Charakters keine Hypothesen formuliert.

### 2.2 Darstellung des bisherigen Wissensstandes

Um dem komplexen Geschehen von Chronifizierungsprozessen bei psychischen Erkrankungen gerecht zu werden, sind die langfristigen Krankheitsverläufe und Behandlungswege (‚Patientenkarrieren’) sowie deren Zusammenhang zu berück­sichtigen: Neben Erkrankungsdauer und Konsultationslatenzen, die in den meisten vorliegenden Analysen untersucht werden (Potreck-Rose & Koch, 1994), sind dabei weitere Faktoren, wie die Art des Krankheitsbeginns und -verlaufs, die Art und Adäquatheit der Vorbehandlungen sowie die Einschätzung der Behandlungsbedarfe durch Patienten und Behandler relevant. Während eine umfassende Analyse, bei der die zuletzt genannten Faktoren berücksichtigt werden, für das deutsche Gesundheitssystem noch aussteht, lässt sich vor dem Hintergrund des ‚fragmentierten’ deutschen psychosozialen Versorgungssystems (Schulz, Barghaan, Harfst & Koch, 2008) schon zum jetzigen Zeitpunkt die Überwindung von Versorgungs­schnittstellen als ein wichtiger Ansatzpunkt zur Optimierung der Behandlungswege von Patienten mit chronischen psychischen Erkrankungen identifizieren. Eine dieser Schnittstellen stellt die Nachsorge nach stationärer Rehabilitation dar. Ziel ist es hierbei, die Effekte der Rehabilitation langfristig zu verstetigen und damit den so genannten rebound-Effekt, d.h. die Reduzierung des Rehabilitationserfolges beim Übergang in den Alltag, zu minimieren: Eigene Ergebnisse auf der Basis umfangreicher Datensätze aus dem Indikationsbereich (Rabung, Mösko, Kawski, Koch & Schulz, 2010) sowie eine umfassende Metaanalyse (Steffanowski, Löschmann, Schmidt, Wittmann & Nübling, 2007) zeigen, dass die Effektstärken zur Katamnese nach nur sechs bzw. zwölf Monaten sich deutlich vermindern und dass damit verbunden auch der Anteil der Rehabilitanden abnimmt, welche statistisch und klinisch signifikant verbessert sind.

Hiermit ist ein zentrales und schwerwiegendes Versorgungsproblem angesprochen. Die Nachsorge selbst sowie die Vorbereitung der und die Empfehlungen zur Nachsorge in der Klinik könnten wichtige Ansatzpunkte für die Überwindung des rebound-Effekts darstellen. Entsprechend kann der Rehabilitationsprozess nicht auf die Zeit der durchschnittlich circa sechswöchigen stationären Behandlung beschränkt sein, sondern es sind weitere Anpassungs- und Bewältigungsprozesse in einer ambulanten Weiterversorgung erforderlich, um eine längerfristige Verstetigung der Behandlungserfolge zu erreichen und einen Transfer der neu gewonnenen Einsichten und Lernerfahrungen in den häuslichen und beruflichen Alltag zu gewährleisten. Dies ist zum Teil auch darin begründet, dass die sozialen Beziehungen und Bindungen, in denen die Beschwerden entstanden sind, in der zumeist initialen stationären Behandlung nur unvollständig bearbeitet und Veränderungen im Handlungsrepertoire nur angebahnt, jedoch nicht stabil verankert werden können. Gerade vor dem Hintergrund der Chronizität der Beschwerden erfordert die In­tegration der neu gewonnenen Handlungs- und Bewältigungskompetenzen in den Alltag langwierigere Anpassungsprozesse. In diesem Sinne zielen die therapeutischen Maßnahmen der stationären Behandlung auf eine Anbahnung und Einübung der nachfolgenden Anpassungs- und Bewältigungsprozesse ab (Harfst, Koch & Schulz, 2002). Die Nachsorgeempfehlungen, die einen obligatorischen Teil der Entlassungsberichte ausmachen, stellen dabei einen zentralen Baustein der Kommunikation an der Schnittstelle stationäre Re­habilitation - ambulante Weiterversorgung/-behandlung dar. Den Entlassungsberichten als Ganzes kommt neben der Funktion als Arztbrief und sozialmedizinischem Gutachten auch eine Vernetzungsfunktion zu, wodurch die stationäre Rehabilitation mit anderen Bereichen des Gesundheitssystems verbunden wird und damit zur Kontinuität in der Versorgungskette beitragen soll.

Eigene KTL-Analysen im Rahmen eines von der DRV geförderten Projekts zur Erstellung von Reha-Therapiestandards bei depressiven Störungen (Barghaan, Koch, Schulz & Dirmaier, Revidierte Version eingereicht; Deutsche Rentenversicherung in Druck) zeigen jedoch, dass in einer Stichprobe von N= 10.599 Rehabilitanden mit depressiven Erkrankungen lediglich 18% mindestens eine Therapieeinheit aus dem Therapiemodul „Nachsorge und soziale Integration“ erhalten haben. Auch eine getrennte Analyse für die einzelnen Rehabilitations­einrichtungen (mit mind. 100 Patienten mit depressiven Störungen) zeigt ein sehr heterogenes Bild: Bei großer Varianz erhielten in den meisten Kliniken weniger als 50% der Patienten mit depressiven Erkrankungen mindestens eine Therapieeinheit aus diesem Modul. Von Experten im Rahmen der Entwicklung der genannten Reha-Therapiestandards als besonders wichtig eingeschätzte Maßnahmen sind dabei die Einleitung spezieller Nachsorgeangebote, die Vermittlung in Selbsthilfegruppen, ambulante Reha-Sportgruppen u. a. sowie Kontakt- und Informationsgespräche mit Vor- und Nachbehandlern.

Neben der Nachsorgevorbereitung in den Kliniken liegen von der Deutschen Rentenversicherung auch erste poststationäre Ansätze vor, um dem beschriebenen Rebound-Effekt entgegen zu wirken. Zu nennen sind hier insbesondere die Nachsorgeangebote „Curriculum Hannover“ und „IRENA“ (Deutsche Rentenversicherung, 2008), die bisher allerdings nicht flächendeckend implementiert sind. Eine wohnortunabhängige Koordination der Nachsorge auf Systemebene, d.h. eine settingübergreifende Koordination und Integration nachfolgender Behandlungen, steht noch aus. Dies bedeutet, dass für den einzelnen Patienten am Heimatort zwar Nachsorgeangebote potentiell vorhanden sind, die von sehr niedrigschwelligen Angeboten, wie z.B. Volkshochschulkursen, über Selbsthilfegruppen, Beratungsstellen und der primärärztlichen Versorgung bis hin zu hochschwelligen Angeboten wie die genannten strukturierten Gruppenangebote, Einzelpsychotherapie oder die fachärztliche Versorgung reichen kann. Eine auf die Situation und die Bedürfnisse des einzelnen Patienten abgestimmte Koordination und ggf. auch Integration dieser potentiell sinnvollen, den Rehabilitationserfolg stabilisierenden Maßnahmen fehlt allerdings noch.

Um diese Lücke zu schließen, bietet sich die Vorhaltung einer patientenorientierten und settingübergreifenden Nachsorge-Koordination an, wobei als konzeptuelle Grundlage für solch ein Angebot die im anglo-amerikanischen Raum verbreitete Methode des Case Management (CM) dienen kann. Unter CM wird ein patientenorientiertes, situationsgeleitetes Betreuungsangebot für Patienten mit schweren oder chronischen Erkrankungen verstanden (Klesse, Bermejo & Härter, 2007), bei dem mit dem Case Manager als ‚Lotsen’ eine koordinierte Behandlungs- und Versorgungsplanung mittels spezifisch definierter Kernkomponenten (Norris, Nicols, Caspersen, Glasgow, Engelgau, Jack et al., 2002) fokussiert wird. So definiert wird deutlich, dass auf einer konkreten Ebene zwischen der genannten Koordination (‚begleitende Fallbetreuung’), d.h. dem Case Management im engeren Sinne und den eigentlichen zu koordinierenden Angeboten, der konkreten Nachsorge vor Ort, zu unterscheiden ist (und erst auf einer abstrakteren Ebene die Koordinations- und Integrationsaufgaben ebenfalls als Teil einer umfassenden Nachsorge verstanden werden können). Systematische Reviews und Metaanalysen zur Wirksamkeit von CM liegen insbesondere für Patienten mit psychotischen Erkrankungen vor (Smith & Newton, 2007). Bezüglich der beiden hier fokussierten Diagnosegruppen Depression und Angsterkrankungen zeigt ein systematisches Review zu CM bei depressiven Erkrankungen nach 6-12 Monaten konsistent positivere Ergebnisse hinsichtlich Symptomatik und Medikamentencompliance im Vergleich zur Standardbehandlung (Gensichen, Beyer, Muth, Gerlach, von Korff & Ormel, 2006). Zu CM bei Angststörungen weisen die vorliegenden kontrolliert-randomisierten Studien ebenfalls auf positive Ergebnisse von CM im Vergleich zur Standardversorgung nach 6-12 Monaten auf Ebene der Symptomatik, Lebensqualität und AU-Zeiten (Rollman, Herbeck Belnap, Mazumdar, Houck, Zhu, Gardner et al., 2005) sowie auf eine Verringerung stationärer Notaufnahmen und der damit verbundenen Kosten hin (Kolbasovsky, Reich, Futtermann & Meyerkopf, 2007). In Deutschland sind CM-Programme für die Indikationsbereiche Depression und Angsterkrankungen bisher nicht verbreitet. Eine erste Effektivitätsüberprüfung eines CM-Modells für Depressive im deutschen Gesundheitssystem liegt von Gensichen et al. (Gensichen, von Korff, Peitz, Muth, Beyer, Güthlin et al., 2009) vor: Diese zeigt, dass telefongestütztes CM in der hausärztlichen Versorgung von depressiven Patienten zu einer stärkeren Symptomreduktion, einer besseren Medikamentencompliance sowie zu einer höheren Patientenzufriedenheit im Vergleich zur Regelversorgung führt. Für den Bereich der Rehabilitation gibt es spezifisch bezogen auf die stufenweise Wiedereingliederung ebenfalls erste positive Erfahrungen eines CM-Modellprojektes (Bürger, 2008).

Erfahrungen und Vorarbeiten zur Durchführbarkeit einer Case Management-Unterstützung sowie zu deren Evaluation (allerdings bei onkologischen Patienten und im Anschluss an die stationäre Akutbehandlung) liegen bei den Antragstellern durch die Bearbeitung des sich in der Abschlussphase befindenden Forschungsprojektes „Case Management in der sektorenübergreifenden Versorgung von Frauen mit Brustkrebs: Effektivität und Effizienz eines optimierten Patientenpfades in einer Modellregion für angewandtes Gesundheitsmanagement“ (BMBF-Förderprogramm Anwendungsorientierte Brustkrebs-forschung) vor (Büscher, Thorenz, Grochocka, Erhardt, Koch & Watzke, 2007), die für das aktuelle Projektvorhaben genutzt werden könnten (insb.: Erfahrungen bei der Implementierung und Durchführung von Case Management orientierten Interventionen, Übertragung der im Projekt zur Anwendung gekommenen Instrumente zur Prozessevaluation).

### 2.3

### a) Ergebnisse der pharmakologisch-toxikologischen Vorprüfungen bei Arzneimittelstudien (§ 40 (1) 5. und 6. AMG)

Entfällt

### b) oder Nachweise über die sicherheitstechnische Unbedenklichkeit des Medizinproduktes sowie Ergebnisse der biologischen Sicherheitsprüfung (§ 20 (1) 6. und 7. MPG)

Entfällt

### c) Handelt es sich um eine Untersuchung, auf die die §§ 23/24 StrahlenschutzVO vom 20.07.2001 Anwendung finden?

Entfällt

### - Werden studienbedingt röntgenologische und /oder nuklearmedizinische Untersuchungen/Behandlungen durchgeführt?

Nein

### 2.4 Begründung der Notwendigkeit von Humanversuchen

### a) Experimente an gesunden Personen?

Entfällt

### b) Heilversuche an Patienten?

Entfällt

### 2.5 Schilderung der geplanten Versuchsausführung einschließlich der statistischen Auswertung

### Studiendesign (Überblick)

Das Projektvorhaben sieht einen kombinierten methodischen Ansatz mit qualitativen und quantitativen Anteilen vor, um einerseits dem komplexen und bisher wenig untersuchten Geschehen von Chronifizierungsprozessen mittels explorativer Analysen gerecht zu werden (Studienteil 1 mit im Schwerpunkt qualitativen Analysen) und andererseits einen konkreten Ansatz zur Verbesserung der Versorgungslage von Patienten mit chronischen psychischen Erkrankungen für die klinische Routine zu evaluieren (Studienteil 2 mit quantitativen Analysen).

**Studienteil 1**

Für Studienteil 1 sollen zunächst in Phase 1 – basierend auf einer zuvor durchgeführten systematischen Literaturanalyse - halbstrukturierte Interviews (Interviewleitfaden siehe Anhang 3) zu den oben aufgeführten Bereichen (a) bis (c) mit Patienten der drei kooperierenden Kliniken (siehe 2.7) mit Depression bzw. Angsterkrankung geführt und qualitativ ausgewertet werden. Hierbei sollen n=36 Patienten (stratifiziert nach Diagnose und Klinik) mit unterschiedlichem Chronifzierungsgrad einbezogen werden. Die Ergebnisse von Phase 1 werden in zweierlei Hinsicht genutzt: Zum einen sollen (vorläufige) Empfehlungen formuliert werden, die u. a. der inhaltlichen Ausgestaltung der NK in Studienteil 2 dienen. Zum anderen soll ein Itemset aus den Interviewergebnissen abgeleitet werden, welches sich für eine schriftliche Befragung einer größeren Patientenstichprobe eignet. Diese schriftliche Befragung schließt sich in Phase 2 des Studienteils 1 an (wird organisatorisch in die t0-Befragung des Studienteils 2 integriert). Entsprechend des Einschlusses von Items mit sowohl offenem als auch geschlossenem Antwortformat beinhaltet die Auswertung der schriftlichen Befragung sowohl qualitative als auch quantitative Anteile.

**Studienteil 2**

Die Ergebnisevaluation zur NK erfolgt im Rahmen eines randomisiert-kontrollierten Designs als prospektive Mehrzeitpunktbefragung einer konsekutiven Patientenstichprobe mit chronischer Angst- oder depressiver Erkrankung in den drei Kooperationskliniken (siehe 2.7): Patienten mit NK (Interventionsgruppe) werden mit Patienten der Regelversorgung (Kontrollgruppe TAU, d.h. ohne NK) hinsichtlich ihres Behandlungsergebnis zu Follow Up verglichen. Das Vorliegen der oben genannten Erkrankungen (F33.x gemäß ICD-10 seit mindestens zwei Jahren oder/und F34.1 bzw. F40.0, F40.1, F41.0 oder/und F41.1 seit mindestens zwei Jahren) wird abgesichert mittels standardisiertem diagnostischen Interview (Mini-DIPS).

Intervention: Patienten werden direkt nach ihrem stationären Aufenthalt mittels einer Nachsorge-Koordination (NK) darin unterstützt, eine für sie adäquate Nachsorge einzuleiten und durchzuführen. Orientierung für das Vorgehen bei der NK bildet der Ansatz des Case Managements. Die NK soll mittels 6 halbstündiger zweiwöchentlich stattfindender Telefonkontakte über einen Zeitraum von 3 Monaten realisiert werden. Es wird von dem jeweiligen Therapeuten durchgeführt, der den Patienten während der Rehabilitation als Bezugstherapeut betreut hat. Im Rahmen der Schulung der Bezugstherapeuten soll diesen zum einen das Vorgehen bei der Nachsorge-Koordination basierend auf der Methode des Case Managements vermittelt werden. Zum anderen sollen die Empfehlungen resultierend aus Studienteil 1 bezüglich der Verbesserung von Steuermechanismen und der Ausgestaltung von Unterstützungs- und Behandlungsangeboten vorgestellt und für die NK umgesetzt werden. Darüber hinaus ist es besonders wichtig, dass die Therapeuten im Rahmen der Schulung Heuristiken an die Hand bekommen, mit deren Hilfe sie Informationen zum Nachbehandlungsangebot im Wohnort des jeweiligen Patienten beschaffen können. Ebenfalls ist darauf zu achten, dass die Therapeuten den multiprofessionellen Ansatz der Rehabilitation auch bei der Nachsorge-Organisation fokussieren, d. h. dass das breite Spektrum an niedrig- und hochschwelligen psychosozialen Unterstützungsmöglichkeiten ausgeschöpft wird.

Messzeitpunkte der Längsschnitterhebung: t0: Beginn der stationären Behandlung; t1: Beendigung der stationären Behandlung/Beginn der NK in der Interventionsgruppe t2: Beendigung der NK bzw. 3 Monate nach t1; t3: Follow-Up (6 Monate nach t2). Die schriftliche Befragung zu t0 und t1 erfolgt innerhalb der Klinik, zu t2 und t3 werden die Patienten postalisch befragt. Die Bearbeitungszeit pro Fragebogenset wird ca. 30 bis 45 Minuten in Anspruch nehmen.

Hauptzielgrößen: (a) gesundheitsbezogene Lebensqualität operationalisiert über Patientenratings anhand des Short Form 8 Health Survey, SF-8 (primary outcome); (b) depressive bzw. ängstliche Symptomatik, Operationalisierung über das Beck-Depressions-Inventar (BDI-II) bzw. das Beck-Angst-Inventar (BAI).

### Statistische Analysen/Auswertung

Die Auswertung der in Studienteil 1 durchgeführten Interviews erfolgt qualitativ gemäß des inhaltsanalytischen Ansatzes von Dey (Dey, 1993; Dey, 1999), bei dem mittels einer mehrstufigen, iterativen Analyse des Datenmaterials ein inhaltlich und logisch konsistentes Kategoriensystem entwickelt und so die Interviewinhalte strukturiert und in Beziehung zu einander gesetzt werden können.

Zur Überprüfung der Hauptfragestellung in Studienteil 2 sollen Vergleiche zwischen der Interventionsgruppe NK und der Kontrollgruppe TAU hinsichtlich der Veränderungen im primären Outcome vorgenommen werden. Hierzu werden univariate Kovarianzanalysen über den Faktor Gruppe (NK/TAU) und Diagnose (Angst/Depression) zu t3 mit t1 als Kovariate zur Kontrolle der Ausgangsbelastung durchgeführt. Gender wird als zusätzliche Kovariate aufgenommen, ebenso die Differenz von t0 zu t1, d.h. das Ausmaß an Veränderung (=Therapieerfolg) während stationärer Behandlung. Zu überprüfen ist der statistische Effekt von Gruppe. Zusätzlich zu per-protocol- sollen intention to treat-Analysen als Sensitivitätsanalysen durchgeführt werden. Die Vorhersage des Outcomes anhand (weiterer) patientenseitiger Prädiktoren wird mittels regressionsanalytischer Verfahren (blockweise lineare Regression) untersucht und anhand des inkrementellen Zuwachs an Varianzaufklärung überprüft.

### 2.6 Literaturangaben

Verwendete Literatur (§ 2.1 - § 2.5)

Barghaan, D., Koch, U., Schulz, H. & Dirmaier, J. (Revidierte Version eingereicht): Entwicklung von Therapiestandards für die Rehabilitation von Patienten mit depressiven Störungen: Analyse von Daten der Klassifikation Therapeutischer Leistungen (KTL).

Bürger, W. (2008): Innovatives Konzept der Stufenweisen Wiedereingliederung der Deutschen Rentenversicherung Rheinland-Pfalz mit begleitender Fallbetreuung im Rahmen der Nachsorge - Indikation, Durchführung und Evaluation. Zwischenbericht.

Büscher, C., Thorenz, A., Grochocka, A., Erhardt, H., Koch, U. & Watzke, B. (2007). *Evaluation of a case management program for women with breast cancer: Conducting a quasi-experimental study within mental health care research.* Paper presented at the IPOS 9th World Congress of Psycho-Oncology, Sept 16-20, 2007, London, UK.

Deutsche Rentenversicherung. (2008). *Empfehlungen zur Weiterentwicklung der Reha-Nachsorge in der Rentenversicherung. Stand 7. April 2008*. Berlin.

Deutsche Rentenversicherung (in Druck). *Methoden- und Abschlussbericht. Reha-Therapiestandards depressive Störungen*. Berlin: DRV.

Dey, I. (1993). *Qualitative Data Analysis. A User-Friendly Guide for Social Scientists*. London: Routledge.

Dey, I. (1999). *Grounding Grounded Theory: Guidelines for qualitative inquiry*. San Diego: Academic Press.

Gensichen, J., Beyer, M., Muth, C., Gerlach, F.M., von Korff, M. & Ormel, J. (2006): Case management to improve major depression in primary health care: A systematic review. *Psychol Med, 36*, 7-14.

Gensichen, J., von Korff, M., Peitz, M., Muth, C., Beyer, M., Güthlin, C., Torge, M., Petersen, J.J., Rosemann, T., König, J. & Gerlach, F.M. (2009): Case management for depression by health care assistants in small primary care practices. *Ann Intern Med, 151*, 369-378.

Harfst, T., Koch, U. & Schulz, H. (2002): Nachsorgeempfehlungen in der psychosomatischen Rehabilitation. Empirische Analysen auf der Basis der einheitlichen Entlassungsberichts der Rentenversicherungsträger. *Die Rehabilitation, 41*, 407-414.

Klesse, C., Bermejo, I. & Härter, M. (2007): Neue Versorgungsmodelle in der Depressionsbehandlung. *Nervenarzt, 78(3)*, 585-596.

Kolbasovsky, A., Reich, L., Futtermann, R. & Meyerkopf, N. (2007): Reducing the number of emergency department visits and costs associated with anxiety: A randomized controlled study. *Am J Managed Care, 13(2)*, 95-102.

Margraf, J. (1994). *Mini-DIPS: Diagnostisches Kurz-Interview bei psychischen Störungen*. Berlin: Springer.

Norris, S.L., Nicols, P.J., Caspersen, C.J., Glasgow, R., Engelgau, M., Jack, L., Isham, J., G., Snyder, S., Carande-Kulis, V., Garfield, S., Briss, P. & McCulloch, D. (2002): The effectiveness of disease and case management for people with diabetes. A systematic review. *Am J Prev Med, 22(4)*, 15-38.

Potreck-Rose, F. & Koch, U. (1994). *Chronifizierungsprozesse bei psychosomatischen Patienten*. Stuttgart: Schattauer.

Rabung, S., Mösko, M., Kawski, S., Koch, U. & Schulz, H. (2010). *Validität meta-analytischer Befunde zur Effektivität stationärer psychosomatischer Rehabilitation. .* Paper presented at the Poster, 61. Arbeitstagung des Deutschen Kollegiums für Psychosomatische Medizin, Berlin, 17.-20. März 2010, Berlin.

Rollman, B.L., Herbeck Belnap, B., Mazumdar, S., Houck, P.R., Zhu, F., Gardner, W., Reynolds, C.F., Schulberg, H.C. & Shear, K. (2005): A randomized trial to improve the quality of treatment for panic and generalized anxiety disorders in primary care. *Arch Gen Psychiatry, 62*, 1332-1341.

Schulz, H., Barghaan, D., Harfst, T. & Koch, U. (2008): Psychotherapeutische Versorgung. *Gesundheitsberichterstattung des Bundes - Heft 41*.

Smith, L. & Newton, R. (2007): Systematic review of case management. *Aust N Z J Psychiatry, 41(1)*, 2-9.

Steffanowski, A., Löschmann, C., Schmidt, J., Wittmann, W.W. & Nübling, R. (2007). *Meta-Analyse der Effekte stationärer psychosomatischer Rehabilitation. Mesta-Studie (Vol. Band 48)*. Bern: Verlag Hans Huber Hogrefe AG.

### 2.7 Bei Multicenter-Studien (LKP in Hamburg):

### a) Ist das Projekt schon in einem anderen Bundesland bei der für den dortigen Prüfarzt zuständigen Ethik-Kommission vorgelegt worden? Wenn ja, bei welcher?

Entfällt. Erstmalige Vorlage.

### b) Wie viele Zentren nehmen teil?

An der Studie sind ein Universitätsklinikum und insgesamt 3 Kooperationspartner beteiligt. Im Einzelnen:

### Studienleitung:

PD Dr. Birgit Watzke & Prof. Dr. Holger Schulz

Institut und Poliklinik für Medizinische Psychologie

Zentrum für Psychosoziale Medizin

Universitätsklinikum Hamburg-Eppendorf

### Kooperationspartner:

MediCilin Seepark Klinik Bad Bodenteich

Sebastian-Kneipp-Str. 1

D-29389 Bad Bodenteich

MediClin Bliestal Klinik

Am Spitzenberg

D-66440 Blieskastel

St. Franziska-Stift

Franziska-Puricelli-Straße 3

D-55543 Bad Kreuznach

### 2.8 Geplanter Studienzeitraum (Beginn / Ende)

Der Beginn der voraus laufenden Datenerhebung in Studienteil 1 (Patienteninterviews) ist voraussichtlich im Sommer 2012. Darauf aufbauend beginnt die Interventionsstudie (Studienteil 2) Herbst 2012. Das Ende der Datenerhebung ist für Ende 2013 anvisiert.

3. Schilderung der voraussehbaren Belastungen und Risiken für die Versuchspersonen

### 3.1 Eingehen auf etwaige Kontraindikationen, Risiken, Ein- und Ausschlusskriterien

**Studienteil 1:** Die in Studienteil 1 vorgesehenen Patienteninterviews sind mit einer Dauer von 40 bis 60 Minuten so konzipiert, dass die Belastung für die Patienten so gering wie möglich ist. Eine Einbettung in den Kontext der Kliniken ist gegeben, falls die Interviews – unerwarteter Weise – z.B. einen erhöhten Gesprächsbedarf (z.B. über das Thema „Bisheriger Behandlungsverlauf“) nach sich ziehen.

**Studienteil 2:** Es sind keine Kontraindikationen für die zu überprüfende Intervention bekannt. Aus voraus laufenden Studien zu Case-Management-orientierten Angeboten für Patienten mit psychischen Störungen kann die Annahme abgeleitet werden, dass die Intervention wahrscheinlich stabilisierende Effekte auf die psychische Gesundheit der Teilnehmer haben wird. Es handelt sich bei der Intervention um ein Angebot, welches im Sinne einer begleitende Beratung verstanden werden kann, und explizit nicht als psychotherapeutisches Nachsorge-Angebot konzipiert ist. Es werden keine Risiken für die einbezogenen Patientengruppen (siehe Ein- und Ausschlusskriterien) erwartet.

**Datenschutz**

Es werden alle erforderlichen gesetzlichen, ethischen und medizinischen Grundsätze der „Good Clinical Practice“ eingehalten. Wichtige Bestandteile der Durchführung sind hierbei: Patientenrekrutierung im Rahmen des informed consent (Die Patienteninformation wurde gemäß des Leitfadens der DRV Bund, Abteilung für Datenschutz, erstellt; bei den Patienten wird ein schriftliches Einverständnis eingeholt); Freiwilligkeit der Teilnahme; Wahrung der Datenschutzbestimmungen, insb. Pseudonymisierung der Daten.

Die in **Studienteil 1** erhobenen Interviews werden auf Tonband aufgezeichnet. Dabei werden keine personenbezogenen Angaben (Name, Geburtsdatum, Adresse oder sonstige Angaben, die Rückschlüsse auf meine Person zulassen) auf Tonband aufgezeichnet. Die Aufzeichnungen dienen ausschließlich zur Unterstützung der Mitschriften und werden nach Abschluss der Auswertung gelöscht. Die Auswertung erfolgt anonymisiert und in aggregierter Form. Die Daten in **Studienteil 2** werden als Patientenselbstratings erhoben, ergänzt um einige Therapeutenangaben, z.B. zur Diagnose. Der Personenbezug der Daten wird direkt nach Datenerhebung in den Kooperationskliniken entfernt, die Zuordnung der Daten zueinander erfolgt ausschließlich über eine Chiffre.

### Ein- und Ausschlusskriterien

Die Einschlusskriterien stellen a) das Vorliegen einer chronischen depressiven Erkrankung, d. h. einer rezidivierenden depressiven Störung (F33.x gemäß ICD-10) seit mindestens zwei Jahren oder/und einer Dystymie (F34.1) oder b) das Vorliegen einer chronischen Angsterkrankung, d.h. einer sozialen (F40.0) oder Agoraphobie (F40.1), einer Panikstörung (F41.0) oder/und einer generalisierten Angststörung (F41.1) seit mindestens zwei Jahren und c) die Teilnahmebereitschaft im Rahmen des informed consent. Das Vorliegen der oben genannten Erkrankungen wird abgesichert mittels standardisiertem diagnostischen Interview (Mini-DIPS; (Margraf, 1994)). Aus­schlusskri­terien sind a) keine ausreichenden deutschen Sprachkenntnisse, b) weniger als drei Tage stationäre Behandlung, c) Teilnahme an einer spezifischen Nachsorge (z.B. „Curriculum Hannover“) oder Fortsetzung einer bereits vor der Rehabilitation begonnenen Psychotherapie.

### 3.2 Abbruchkriterien (individuell und für die Gesamtstudie)

Studienteil 1: Die in Studienteil 1 durchgeführten Interviews werden bei schädigender Wirkung auf den Patienten abgebrochen.

Studienteil 2: Wird im Rahmen der telefonischen NK zwischen Bezugstherapeut und einem Patienten deutlich, dass die NK eine schädigende Wirkung auf den Patienten hat, soll von einer weiteren Teilnahme des Patienten an der telefonischen NK abgesehen werden. Diese Entscheidung soll auf Basis eines persönlichen Gespräches mit dem Patienten und möglichst im beidseitigen Einvernehmen getroffen werden. Die Gesamtstudie soll abgebrochen werden, wenn sich die Mehrheit der Teilnehmer unter der telefonischen NK deutlich klinisch verschlechtert.

4. Darlegung der voraussichtlichen Vorteile und der Bedeutung des Versuches für den Menschen

### a) in der Heilkunde (namentlich unter Vergleich mit herkömmlichen diagnostischen resp. therapeutischen Methoden)

Ergebnisse aus Studienteil 1 können Ansatzpunkte für eine an den Erfahrungen, Bedarfen und Wünschen der Rehabilitanden orientierte Optimierung von Behandlungs-/Unterstützungsangeboten und Steuer­mechanismen im Versorgungssystem liefern.

Hinsichtlich des Studienteils 2 würde bei entsprechend positiven Evaluationsergebnissen mit telefonbasierter NK ein umsetzbares, effektives Modell zur wohnortunabhängigen Nachsorge für die Routineversorgung der Rentenversicherung zur Verfügung gestellt werden. Zusätzliche gender- und diagnosespezifische Effektivitätsdaten würden ebenfalls vorliegen. Bei erfolgreicher Einführung der NK erscheint, neben einer flächendeckenden Einführung für die beiden hier untersuchten Diagnosegruppen, eine Übertragung auf Patientengruppen mit anderen psychischen Erkrankungen, ggf. auch auf Rehabilitationspatienten mit anderen chronischen Erkrankungen, sinnvoll.

Ein telefonbasiertes Angebot zur Vermittlung und Koordination von Nachsorgemaßnahmen nach stationärer Rehabilitation bringt vor dem Hintergrund der gesundheitspolitischen Bedeutung folgende Vorteile mit sich: a) Durch die räumliche „Unabhängigkeit“ des Angebots besteht die Möglichkeit, dass die Nachsorge-Koordination von dem vorher behandelnden Therapeuten durchgeführt wird (hierdurch: Sicherstellung der Behandlungskontinuität; Nutzung der bereits bestehenden therapeutischen Beziehung und des Vorwissens des Therapeuten über den Patienten im Sinne des Bezugstherapeutenkonzepts). Darüber hinaus entfallen Fahrzeiten / Fahrtkosten. b) Das Angebot kann als patientenorientiertes Steuerinstrument eine settingübergreifende Nachsorge vermitteln und bei dem Umgang mit versorgungsbedingten (z.B. Wartezeiten; Intransparenz vorgehaltener Angebote) sowie patientenseitigen (z.B. Ängste; fehlende Motivation) Barrieren unterstützen und diesen begegnen (u.a. Versorgungsalternativen/Überbrückungs­möglichkeiten aufzeigen, wie z.B. Selbsthilfe; Internet-Angebote; Copingstrategien zum Umgang mit Ängsten auffrischen; Motivationsanalyse/-aufbau leisten).

### b) in der Forschung

Aus den Ergebnissen der qualitativen Analysen aus Studienteil 1 soll ein konzeptueller Beitrag zur Weiterentwicklung des komplexen Forschungsgebiets der Chronifizierungsprozesse bei psychischen Erkrankungen geleistet werden. Darüber hinaus wird ein Itemset zur schriftlichen Erhebung und Dokumentation von Chronifizierungsprozessen, Patientenwegen und -bedarfen entwickelt, welches sich für eine weitere Beforschung des Feldes, aber auch für den Einsatz in der Versorgungsroutine eignet.

Mit den aus Studienteil 2 erzielten Ergebnissen können Erkenntnisse zur Akzeptanz und der Effektivität einer niedrigschwelligen Nachsorge-Koordination gewonnen werden. Die Untersuchung eines telefonischen Angebots ermöglicht darüber hinaus die Erweiterung des empirischen Wissens zu einem Bereich, der im weiteren Sinne den Neuen Medien zuzurechnen ist und zu dem bisher wenige systematische wissenschaftliche Studien vorliegen.

### Besondere Überlegungen sind bei Randomisierung, Verblindung und Placebo-Kontrollen angesichts der ungleichen Behandlung der Gruppen nötig (u.a.: Ist die Vorenthaltung einer aussichtsreichen neuartigen Behandlung gegenüber der Kontrollgruppe vertretbar? Gibt es eine Standardtherapie?)

Bei der zu untersuchenden Intervention handelt es sich um ein die Routineversorgung ergänzendes Angebot. Die Überprüfung der Effektivität einer telefonbasierten NK nach stationärer Psychotherapie bei Depressions- bzw. Angstpatienten erfolgt nach Kenntnisstand der Antragsteller erstmalig, so dass offen bleibt, inwieweit ein zusätzlicher Nutzen im Sinne der Verbesserung oben genannter Ergebniskriterien (siehe 2.5) für die behandelten Patienten entsteht.

### 4.1 Bei minderjährigen gesunden Versuchspersonen (vgl. dazu § 40 (1) 1.-3. und (4) 1.-4.) AMG bzw. § 20 (4) MPG)

### a) Spezieller Bezug zu Krankheiten von Minderjährigen?

Entfällt

### b) Individuelle Indikation?

Entfällt

### c) Möglichkeit der Prüfung an Erwachsenen ausgeschlossen?

Entfällt

### 4.2 Bei Patienten als Versuchspersonen: Individuelle Indikation?

Es soll eine konsekutive Stichprobe von chronifizierten Angst- und depressiven Patienten eingeschlossen werden.

### 4. 3 In Abschnitt 4 verwendete Literatur

Entfällt.

5. Güterabwägung zwischen den Nachteilen und Risiken einerseits und dem voraussichtlichen Nutzen andererseits

Die Effektivität der zu überprüfenden telefonbasierten Nachsorge-Koordination für Patienten mit chronifizierten Angsterkrankungen und Depressionen ist noch nicht nachgewiesen. Die Standardbehandlung sieht keine spezifische, systematisierte Nachsorge-Koordination vor.

### 5.1 Berücksichtigung des Grundsatzes, dass stets die Belange der Versuchsperson den Vorrang haben müssen (vgl. dazu Ziff. I 5, 6, II. 4 und 6 der revidierten Deklaration von Helsinki; § 40 (1) 1. AMG, § 20 (1) 1. MPG)

Die Planung und Durchführung dieser Untersuchung wird eindeutig und umfassend in einem Versuchsprotokoll niedergelegt. Die Forschungsgruppe im Institut und Poliklinik für Medizinische Psychologie, Zentrum für Psychosoziale Medizin, Universitätsklinikum Hamburg-Eppendorf und alle im Projekt kooperierenden Beteiligten folgen der Pflicht, die Ethik-Kommission über den Versuchsablauf zu informieren, insbesondere über alle während des Versuchs auftretenden ernsten Zwischenfälle.

Mit der Durchführung und Auswertung der Studie sind ausschließlich wissenschaftlich qualifizierte Personen beauftragt. Die telefonbasierte Nachsorge-Koordination wird jeweils von klinisch kompetenten, psychotherapeutisch ausgebildeten Person durchgeführt. Die Verantwortung für die Versuchsperson trägt stets eine psychotherapeutisch qualifizierte Person und nie die Versuchsperson selbst, auch dann nicht, wenn sie ihr Einverständnis gegeben hat. Das Wohlergehen der Versuchsperson hat stets Vorrang vor den Interessen der Wissenschaft und der Gesellschaft.

### 5.2 Beschränkung der Zahl der Versuchspersonen auf das unbedingt notwendige Maß (einschließlich begründeter biometrischer Berechnungen)

Studienteil 1: In jeder der drei Kliniken werden jeweils sechs Patienten der beiden Indikationsbereiche (Depression und Angsterkrankungen) befragt, so dass insgesamt 36 Patienten interviewt werden. Diese Stichprobengröße erscheint notwendig und angemessen vor dem Hintergrund, dass hiermit für beide Erkrankungsgruppen Patienten mit sowohl positiv als auch negativ verlaufenden Behandlungswegen befragt werden können und dass somit ein umfassendes Spektrum an Patientenerfahrungen in der Studie erfasst werden kann.

Studienteil 2: Es wird eine Stichprobengröße angestrebt, bei der mit einer statistischen Power von 80% ein kleiner Effekt zwischen NK und TAU in den geplanten ANCOVA nachgewiesen werden kann. Wir definieren eine kleine Effektstärke als eta^2^=0.02 (bzw. f=0.14 gemäß Cohen). Angenommen wird, dass die Fehlervarianz sich um 30% aufgrund des Einschlusses der Ausgangsbelastung t1 als Kovariate (welche aufgrund der Randomisierung als nicht korreliert zu den Untersuchungsbedingungen anzusehen ist) reduziert. Dieses führt zu einer adjustierten Effektgröße von eta^2^=0.03 (f=0.17). Bei alpha=0.05 ist somit eine Stichprobengröße von insgesamt n=274 Patienten (pro Untersuchungsbedingung n=137) zu erreichen. Erfahrungen aus voraus laufenden Projekten der Antragsteller lassen eine Drop-out-Quote von 30% realistisch erscheinen, so dass mit Berücksichtigung dieser Ausfallquote 196 Patienten pro Unter­su­chungs­­bedingung rekrutiert werden sollen. Abbildung 1 gibt im Überblick Design, Stadien und Stichprobengröße des Forschungsprojekts wieder.

Abbildung 1. Design Studienteil 2

**Stationäre Rehabilitation**

**Screening von**

**ca. 2.760 Patienten**

**Interventionsgruppe telefonbasierte**

**Nachsorge-Koordination**

**n=196**

**Kontrollgruppe TAU**

**ohne spezifische**

**Nachsorge-Koordination**

**n=196**

**TAU**

**n=137**

**t_0_**

**t_1_**

**t_2_**

**t_3_**

**NK**

**n=137**

**Follow-up**

**Drop out: ca. 30%**

**3 Monate**

**6 Monate**

**Patienten**

**gemäß Ein- und Ausschlusskriterien**

**ca. n=1380**

**Teilnehmer**

**n=392**

**(bei einer**

**Teilnahmequote**

**von ca. 30%)**

### 5.3 Bei Versuchen an gesunden Probanden, denen keine therapeutischen Vorteile aus dem Versuch erwachsen, gelten strengere Anforderungen an die Vertretbarkeit des Forschungsvorhabens als bei neuartigen Heilversuchen an Patienten (vgl. die unterschiedlichen Anforderungen in § 40 AMG gegenüber § 41 AMG)

Entfällt

### 5.4 Besondere Überlegungen sind bei Blind- und Doppelblindversuchen an Patienten angesichts der ungleichen Behandlung der beiden Gruppen nötig (u.a.: ist die Vorenthaltung einer aussichtsreichen neuartigen Behandlung gegenüber der Kontrollgruppe vertretbar?)

Siehe Abschnitt 4.

6. Angaben über den Inhalt der Aufklärungsgespräche mit den Versuchspersonen

In beiden Studienteilen werden diejenigen Patienten, welche die Einschlusskriterien der Studie erfüllen, durch das Klinikpersonal informiert und aufgeklärt. Zusätzlich erhalten sie schriftliches Material mit Informationen zur Studie. Patienten werden im Rahmen des *informed consent* um die Teilnahme an der Studie gebeten. In Studienteil 2 erfolgt die Information über die Gruppenzugehörigkeit (NK und TAU) in der letzten Woche durch den Bezugstherapeuten, um eine unterschiedliche Behandlung während der stationären Rehabilitationsbehandlung resultierend aus dem Wissen über die Gruppenzugehörigkeit eines Patienten zu vermeiden.

### 6.1 Bei Patienten / Probanden

### a) Diagnoseaufklärung

Entfällt.

### b) Alternative Verlaufserklärung (Prognoseaufklärung)

Entfällt.

### c) Risikoaufklärung, bezogen auf die Versuchsauswirkungen

Eine Risikoaufklärung soll nicht erfolgen, da aufgrund von vorausgehenden Studien nicht von einer erhöhten Risikolage auszugehen ist.

### d) Aufklärung über ein angebrachtes Verhalten der Versuchsperson während des Versuches und nach dem Versuch

Entfällt.

### e) Aufklärung über die Aufzeichnung von Krankheitsdaten und deren Weitergabe zur Überprüfung an den Auftraggeber, die zuständige Überwachungsbehörde (gemäß § 67 AMG) oder die zuständige Bundesoberbehörde (§ 40 (1) 2. AMG; § 20 (1) 2. MPG

Patienten werden im Rahmen der Informationsvermittlung über die Studie darüber informiert, dass die Angaben aus den Interviews, den Fragebögen, dem telefonischen Kontakt und die medizinischen Basisdaten in anonymisierter Form und unter Wahrung der Bestimmungen des Datenschutz für fünf Jahre nach Beendigung der Studie gespeichert werden. Der Förderer, d.h. das BMBF und die DRV-Bund, erhält keine patientenbezogenen Daten, auch nicht anonym, sondern nur Auswertungen in aggregierter Form als Bericht.

### f) Studienbedingte röntgenologische / nuklearmedizinische Untersuchungen

Entfällt

### 6.2 Aufklärung über die Widerruflichkeit einer Einwilligung?

Ist in Einwilligungserklärung enthalten (siehe Abschnitt 7).

### 6.3 Besondere Aufklärung über die Situation

### a) bei der randomisierten Studie

siehe Abschnitt 7.

### b) beim Blind- und Doppelblindversuch

Entfällt

### c) bei placebo-kontrollierten Studien

Entfällt

### 6.4 Beachtung der Sonderregeln bei Minderjährigen und bei in ihrer Geschäftsfähigkeit beeinträchtigen Patienten (§ 40 (4) 1.-4. und § 41 2.-7. AMG; § 17 (4) 4. und § 20 (4) MPG)

Entfällt

7. Beifügung eines Musters für die (schriftlich) zu erteilende Aufklärung sowie die Einwilligungserklärung (vgl. § 40 (2) 2. AMG; § 20 (1) 2. MPG)

Liegen dem Antrag in generischen, d.h. klinikunspezifischer, Form bei (siehe Anhang 1 und 2). Die jeweiligen Anpassungen (z.B. Klinik-Briefkopf, Anrede, etc.) sind nicht inhaltlicher Natur. *(Aktuell liegen diesem Antrag die Materialien für Studienteil 1 bei, für Studienteil 2 sind die Materialien in der Erstellungsphase.)*

8. Nachweis einer ausreichenden Probandenversicherung

### 8.1 Aufklärung über das Bestehen und den Umfang einer Probandenversicherung (§ 40 (1) 8. AMG; § 20 (1) 9. MPG) und die danach von der Versuchsperson zu beachtenden Obliegenheiten im Falle des vermuteten Schadens und im Todesfall (s. Allgemeine Versicherungsbedingungen

### - für klinische Prüfungen von Arzneimitteln

### - für klinische Prüfungen von Medizinprodukten nach dem MPG)

Von einer eigens für klinische Studien abzuschließende Probandenversicherung kann abgesehen werden, da es sich nicht um die Anwendung eines Arzneimittels, bzw. eines unter das MPG fallendes Medizinprodukt handelt. Die dem Forschungsprojekt eigenen Gefahren für den Probanden und der Schutz des Studien- Leiters sowie der Bezugstherapeuten werden durch die Betriebshaftpflichtversicherung des jeweiligen Klinikums abgedeckt.

9. Darlegung der Erfüllung etwaiger sonstiger Voraussetzungen für die Durchführung des Vorhabens

(z.B. § 40 (1) 3., 6., 7. AMG, § 67 (1) AMG)

Entfällt

10. Maßnahmen bei Veränderung der Risikolage

### 10.1. Sicherstellung, dass bei Veränderungen der Risikolage während des Versuches die Güterabwägung im Sinne von Ziffer 5. jeweils erneut durchgeführt wird und bei nachteiliger Veränderung der Risikolage auch erneute Aufklärungsgespräche mit den Versuchspersonen stattfinden

Wird gewährleistet.

### 10.2. Mitteilung der veränderten Abbruchkriterien an die Ethik-Kommission

Wird gewährleistet.

### 10.3. Mitteilung schwerwiegender oder unerwarteter Ereignisse an die Ethik-Kommission.

Wird gewährleistet.


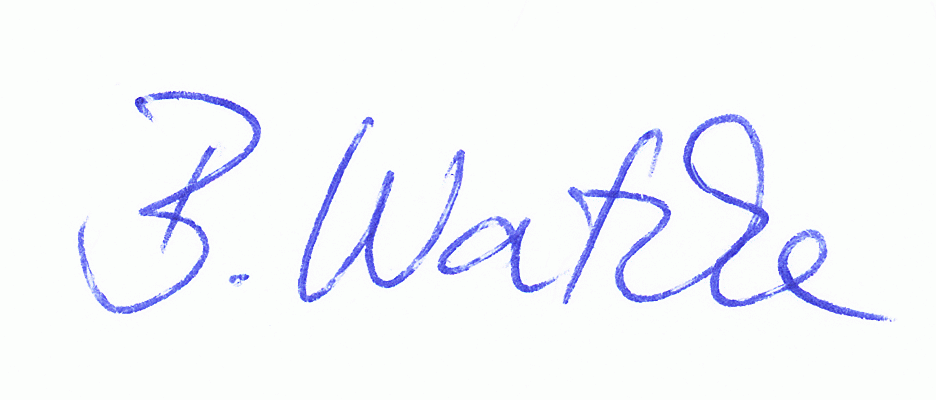


Hamburg, den 18.11.2011 _________________________

PD Dr. Birgit Watzke

Studienleitung
