# Supplementary material for: Effectiveness of telephone-based aftercare case management for adult patients with unipolar depression compared to usual care: A randomized controlled trial
Source: PLoS One. 2017 Oct 27;12(10):e0186967. doi: 10.1371/journal.pone.0186967 (PMC5659793; doi:10.1371/journal.pone.0186967)
Supplement: S1 Translation relevant parts clinical trial protocol — (DOCX) [file pone.0186967.s008.docx]

### [...]

### 2.5 Study design (Overview)

Intervention (page 7): The aftercare coordination is based on the concept of case management, which is a patient-centered approach that aims to support the patient in finding and organizing his or her individual aftercare treatment. After the inpatient treatment, the patients in the intervention group will receive six aftercare coordination phone contacts at intervals of two weeks that will be performed by their inpatient treatment therapists for 12 weeks. Prior to initiating the aftercare coordination, the therapists will be trained in their role as a coordinator and receive an elaborate manual that will provide guidelines for the phone contacts. This detailed manual contains helpful instructions for the therapist but allows sufficient freedom to tailor the coordination to the patients’ needs, individual situations and conditions. The contents of the manual include descriptions of the aims of the aftercare coordination, the processes of the study and the phone contacts and instructions for dealing with specific situations that are demonstrated via examples.

[...]

Outcomes^[[1]](#footnote-1)^: (a) health-related quality of life as measured with the Short Form 8 Health Survey, SF-8 (primary outcome); (b) depressive and anxiety symptoms, assessed using Beck’s Depression Inventory (BDI-II) and Beck Anxiety Inventory (BAI).

[...]

### 3.1. Inclusion and exclusion criteria (page 11)

Inclusion criteria are a) a diagnosis of a chronic depressive disorder (F32.x, F33.x, F34.1, according ICD-10) since at least two years or b) a diagnosis of anxiety (F40.x, F41.x, according to ICD-10) since at least two years and c) willingness to participate within informed consent. The diagnoses will be validated with a diagnostic interview (Mini-DIPS; (Margraf, 1994)). Exclusion criteria are a) insufficient German language skills, b) an inpatient treatment duration of less than three days, c) Participation in a specific aftercare programme (e.g. „Curriculum Hannover“) or continuation of a concurrent outpatient psychotherapeutic treatment already started before admission.

[...]

### 6. Information regarding the recruitment and informed consent discussion with the participants (page 14)

The patients who fulfill the study inclusion criteria are recruited and informed by the clinical staff at the beginning of their inpatient treatment. Prior to participation, the patients are informed with oral and written information regarding the study by their therapists. After written informed consent is obtained, the patients will be randomly assigned to the intervention group or the TAU group. The therapists will be informed about the randomization outcome in the last week of inpatient treatment so that they can inform the patients about their group assignment before discharge.

1. The original primary outcome (quality of life) was changed at an early stage before trial registration. As described in the study registration (clinicaltrials.gov) and the published study protocol, the current primary outcome is symptom severity. [↑](#footnote-ref-1)
